# A bead-based multiplex assay covering all coronaviruses pathogenic for humans for sensitive and specific surveillance of SARS-CoV-2 humoral immunity

**DOI:** 10.1038/s41598-023-48581-9

**Published:** 2023-12-09

**Authors:** Daniel Stern, Tanja C. Meyer, Fridolin Treindl, Hans Werner Mages, Maren Krüger, Martin Skiba, Jan Philipp Krüger, Christian M. Zobel, Maximilian Schreiner, Marica Grossegesse, Thomas Rinner, Caroline Peine, Anna Stoliaroff-Pépin, Thomas Harder, Natalie Hofmann, Janine Michel, Andreas Nitsche, Silke Stahlberg, Antje Kneuer, Anna Sandoni, Ulrike Kubisch, Martin Schlaud, Annette Mankertz, Tatjana Schwarz, Victor M. Corman, Marcel A. Müller, Christian Drosten, Kathrin de la Rosa, Lars Schaade, Martin B. Dorner, Brigitte G. Dorner

**Affiliations:** 1 Biological Toxins (ZBS 3), Centre for Biological Threats and Special Pathogens, Robert Koch Institute, 13353 Berlin, Germany; 2 Department of Microbiology and Hospital Hygiene, Bundeswehr Hospital Berlin, Berlin, Germany; 3 Department of Internal Medicine, Bundeswehr Hospital Berlin, Berlin, Germany; 4 Highly Pathogenic Viruses (ZBS 1), Centre for Biological Threats and Special Pathogens, Robert Koch Institute, 13353 Berlin, Germany; 5 Immunization Unit (FG 33), Department for Infectious Disease Epidemiology, Robert Koch Institute, 13353 Berlin, Germany; 6 Central Epidemiological Laboratory (FG 22), Department of Epidemiology and Health Monitoring, Robert Koch Institute, 12101 Berlin, Germany; 7 Measles, Mumps, Rubella, and Viruses Affecting Immunocompromised Patients (FG 12), Robert Koch Institute, 13353 Berlin, Germany; 8 Institute of Virology, Charité-Universitätsmedizin Berlin, 10117 Berlin, Germany; 9 Corporate Member, Freie Universität Berlin, 10117 Berlin, Germany; 10 Corporate Member, Humboldt-Universität zu Berlin, 14195 Berlin, Germany; 11 Max-Delbrück-Center for Molecular Medicine in the Helmholtz Association (MDC), 13125 Berlin, Germany; 12 Berlin Institute of Health (BIH), Charité-Universitätsmedizin Berlin, 10117 Berlin, Germany; 13 Centre for Biological Threats and Special Pathogens, Robert Koch Institute, 13353 Berlin, Germany

**Keywords:** Biochemical assays, SARS-CoV-2, Infectious diseases, Viral infection, Diagnostic markers, Viral infection, Viral proteins, Immunological techniques, ELISA

## Abstract

Serological assays measuring antibodies against SARS-CoV-2 are key to describe the epidemiology, pathobiology or induction of immunity after infection or vaccination. Of those, multiplex assays targeting multiple antigens are especially helpful as closely related coronaviruses or other antigens can be analysed simultaneously from small sample volumes, hereby shedding light on patterns in the immune response that would otherwise remain undetected. We established a bead-based 17-plex assay detecting antibodies targeting antigens from all coronaviruses pathogenic for humans: SARS-CoV-2, SARS-CoV, MERS-CoV, HCoV strains 229E, OC43, HKU1, and NL63. The assay was validated against five commercial serological immunoassays, a commercial surrogate virus neutralisation test, and a virus neutralisation assay, all targeting SARS-CoV-2. It was found to be highly versatile as shown by antibody detection from both serum and dried blot spots and as shown in three case studies. First, we followed seroconversion for all four endemic HCoV strains and SARS-CoV-2 in an outbreak study in day-care centres for children. Second, we were able to link a more severe clinical course to a stronger IgG response with this 17-plex-assay, which was IgG1 and IgG3 dominated. Finally, our assay was able to discriminate recent from previous SARS-CoV-2 infections by calculating the IgG/IgM ratio on the N antigen targeting antibodies. In conclusion, due to the comprehensive method comparison, thorough validation, and the proven versatility, our multiplex assay is a valuable tool for studies on coronavirus serology.

## Introduction

Since the first reports of a novel respiratory disease in the city of Wuhan broke the news at the end of 2019, the worldwide pandemic caused by the novel coronavirus SARS-CoV-2 has led to an unprecedented global health crisis, mirrored in modern times only by the notorious pandemic Spanish Flu from 1918^1^. Despite recent progress in fighting back SARS-CoV-2 based on a rapidly progressing worldwide vaccination campaign and accruing herd immunity, SARS-CoV-2 is here to stay as an endemic virus that has to be monitored much alike seasonal influenza^2^. This development is driven by an ongoing number of SARS-CoV-2 variants (variants of concern or VOCs: Alpha, Beta, Gamma, Delta, Omicron, etc.) able to escape the immune response. This leads to novel infections with variants, although with a lower impact on public health due to previous contact through either immunisation and/or infection. Hence, specific and sensitive assays to monitor correlates of alleged immunity against SARS-CoV-2 with the ability to be adaptable to novel variants are needed. Such assays are useful for surveillance and monitoring of the longitudinal immune response induced by both natural infections and vaccination programs today and in the future.

The *Coronaviridae* family contains diverse virus species able to infect both animals and humans, hereby exhibiting a high potential for zoonotic infections^3^. Of these, seven members are able to infect humans and are assigned to the highly pathogenic zoonotic coronavirus strains SARS-CoV, SARS-CoV-2, and MERS-CoV, and the endemic human pathogenic coronavirus (HCoV) strains HCoV-HKU1, HCoV-OC43, HCoV-NL63, and HCoV-229E^4, 5^. Regarding the humoral immune response against coronaviruses after infection, antibodies against the spike (S) or nucleocapsid (N) proteins are most abundant and consequently most frequently used for detection of antibodies^6, 7^. Here, antibodies targeting the S protein and more specifically the receptor binding domain (RBD) located on the S1 subunit of the S protein mediate protection by inhibiting receptor binding^8^. Therefore, the S protein is also the antigen of choice for most vaccines targeting SARS-CoV-2^9^. Conversely, antibodies targeting the N protein are not induced by immunisation with S-specific vaccines but can be found after infection with SARS-CoV-2^10–12^. Therefore, N-specific antibodies can be used for discrimination of vaccinated and recovered individuals, respectively.

The immune response against coronaviruses and especially SARS-CoV-2 has been studied extensively in the previous years. Here, all arms of the immune response—innate^13^, cellular^14^, and humoral^15^—have been put under scrutiny to answer crucial questions about mechanisms contributing to the pathology of SARS-CoV-2^16, 17^ as well as the induction, endurance^18^, and breadth of protection against wildtype SARS-CoV-2 and different VOCs induced by both infection^19^ and immunisation^12, 20, 21^. Serologic assays play a pivotal role in answering these questions^22–24^. By quantifying binding of antibodies to different viral proteins, questions about the induction of a long-lasting immune response after vaccination by measuring S-specific IgG antibodies^25^, reactivity and neutralisation of antibodies against VOCs^26^, specific detection of natural infections by measuring N-specific IgG antibodies, or the time since past infections by determining the IgA, IgM and/or IgG response can be addressed^27^. Furthermore, assays measuring antibody binding to the RBD showed good correlation with the level of neutralising antibodies as determined by cell culture-based virus neutralisation (VNT) assays^28^ or surrogate virus neutralisation test (sVNT) measuring binding inhibition between the RBD and recombinant ACE-2 receptors^29, 30^. Hence, such assays can be used as a proxy to determine the level of neutralising antibodies in a patient’s sample^31^.

To this aim, a plethora of both commercial and in-house immunoassays have been developed^32^. Of those, multiplex assays, addressing several antigens simultaneously, offer various advantages over single-plex assays. First, by combining different SARS-CoV-2 specific antigens, the antibody immune response induced after vaccination can be addressed side-by-side with the antibody immune response after infection by combining S and N proteins^33^. Second, by including viral antigens of other coronaviruses, the role of cross-reactive antibodies can be assessed and considered. Third, multiplexing allows for the inclusion of internal assay controls such as positive or negative controls^34–36^. Additionally, as only a small sample volume is needed, multiplex assays are ideally suited for studies with infants or children where sample material is often limited^37^. Finally, multiplex assays enable flexible setups depending on the research question addressed as well as detection of different antibody isotypes^38^ and subclasses^39^ from various sample materials such as serum, plasma^40^, dried blood spots, or sputum^38^.

In this work we describe the development and validation of a bead-based serological multiplex assay for detection of IgG and IgM antibodies against all human coronaviruses. To this aim, four SARS-CoV-2 specific antigens—full-length trimeric spike protein, the S1 and its RBD domain as well as the N protein—were coupled to individually colour-coded paramagnetic xMAP^®^ beads. SARS-CoV-2 specific beads were combined with beads coupled with recombinant S1 domains from SARS-CoV, MERS-CoV and RBD domains from the four endemic HCoV-strains HKU1, OC43, NL63, and 229E. The assay was thoroughly validated by method comparison with six commercial assays in addition to a cell-based VNT assay on sera sampled during serological studies in SARS-CoV-2 outbreak hotspots in Germany in 2020^41^ compared to pre-pandemic sera. Special emphasis was laid on setting robust and meaningful cut-off values to discern positive from negative samples.

Finally, we showed the broad adaptability of the multiplex assay to multiple research questions in four applications: first, by detecting antibodies from both serum and eluted dried blood spots (DBS), second, by analysing both the antibody immune response against HCoVs and SARS-CoV-2 during outbreaks in day-care centres. Third, we applied our assay in a clinical study correlating disease severity of patients hospitalised due to COVID-19 with seroconversion rates. We were able to show that severe COVID-19 was not only associated with earlier and more complete seroconversion: by integrating IgG-isotype-specific detection antibodies we could show that the humoral immune response is dominated by an IgG1 and—to a lesser degree—by an IgG3 immune response. Lastly, we used the ability of our multiplex assay to differentiate antibodies induced by infection from antibodies induced by vaccination based on S and N protein reactivity in conjunction with IgG and IgM detection. Hereby, we were able to reliably discriminate recent from previously unrecognized SARS-CoV-2 infections that might otherwise impact the study of vaccine efficacy if not accounted for. The highly flexible and modular setup together with a thorough validation allowed us to develop a versatile multiplex-suspension assay for detection of IgG and IgM antibodies against all known human coronaviruses.

## Results

### SARS-CoV-2 specific antibodies can be detected with high specificity and sensitivity

#### Characterisation of and rational for the antigens used

**Figure 1:**
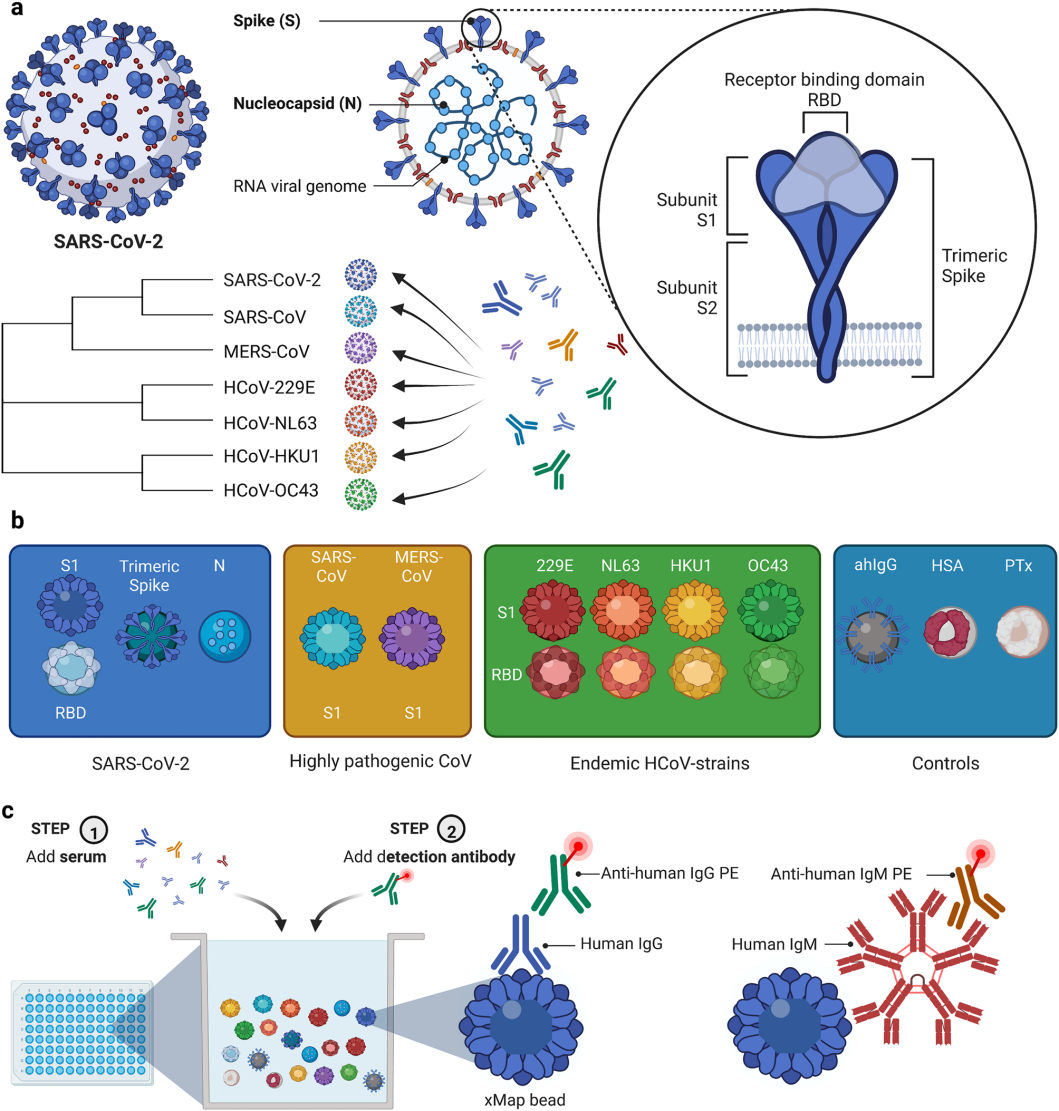
Coronaviral antigens and setup of the multiplex-suspension assay for specific detection of the humoral IgG and IgM response against SARS-CoV-2 and other coronavirus-strains pathogenic to humans. (**a**) Schematic structure of SARS-CoV-2 virions with the two major targets of the humoral immune response, the spike (S) and nucleocapsid (N) proteins. Different domains of the trimeric S protein can be discerned with the receptor binding domain (RBD) within the highly specific S1 subunit. The subunit S2 was explicitly not targeted in our assay since it is more conserved within the coronavirus family hereby potentially leading to a higher degree of cross-reactivity between members of the coronavirus family. Seven coronavirus strains comprising highly pathogenic strains (SARS-CoV-2, SARS-CoV, MERS-CoV) as well as endemic HCoV-strains (229E, NL63, HKU1, OC43) are able to infect humans hereby inducing a humoral antibody response. (**b**) Antigens implemented in the 17-plex multiplex assay comprised four SARS-CoV-2 specific targets, the S1 and RBD domains, trimeric spike protein, as well as the nucleocapsid protein (N), the S1 domains from SARS-CoV and MERS-CoV, both the S1 and RBD domains of the four endemic HCoV-strains as well as three controls, anti-human IgG (ahIgG), human serum albumin (HSA), pertussis toxin (PTx), all coupled to different colour-coded xMAP^®^-beads (Luminex^®^). (**c**) Setup and experimental procedure of the multiplex-suspension assay. To a bead-mix comprising all 17 different bead regions diluted serum or blood eluted from dried blood spots (DBS) is added. Antibodies bound to the antigens immobilised to the xMAP^®^ beads are subsequently detected using an anti-human IgG antibody coupled to phycoerythrin (PE) or an anti-human IgM antibody coupled to PE. Figure created with BioRender.com.

The aim of this work was to establish a highly sensitive and specific bead-based multiplex assay not only for large-scale population-based studies, but also for specific research questions with regard to SARS-CoV-2 induced humoral correlates of alleged immunity. Therefore, a variety of commercially available and in-house produced antigens comprising the highly pathogenic SARS-CoV-2, SARS-CoV, and MERS-CoV, as well as the endemic human coronaviruses characterised by lower pathogenicity, HCoV-HKU1, HCoV-OC43, HCoV-NL63, and HCoV-229E were included (Fig. 1, Table S1, Fig. S1). For the highly pathogenic coronaviruses SARS-CoV and MERS-CoV, only the S1 domains were commercially available and hence used in our assay while for the endemic HCoV strains both the S1- and the RBD-domains were obtained from commercial suppliers or recombinantly expressed successfully in-house for integration in our assay. Finally, four antigens were targeted for detection of the SARS-CoV-2 specific antibody response: a recombinant trimeric spike protein (TriS), a recombinant S1 and RBD domain as well as the full-length N protein. We used these multiple antigens to address multiple questions. First, by including the RBD and/or the S1 domains from all human pathogenic coronaviruses we wanted to test for potential cross-reactive antibody responses to ensure maximum specificity of the SARS-CoV-2 specific antibody response. Second, by targeting the S protein of SARS-CoV-2 in three different forms (TriS, S1, RBD) we aimed at measuring potentially neutralising antibodies, but also non-neutralising antibodies with a target outside the receptor binding site on the S protein^15^. Finally, besides the S protein, the N protein was included as a second immunodominant protein and as a potential marker for differentiation between infection and vaccination. Since the vaccines used in the Western Hemisphere are mostly based on recombinant S proteins^42^, vaccination with S-based vaccines leads to production of antibodies targeting the S protein exclusively while infections potentially induce antibodies directed against both the S and N protein. Additionally, human serum albumin was integrated as a negative control antigen while goat anti-human IgG was used as a positive control to test for the presence of IgG antibodies in the given blood-derived samples. Finally, pertussis toxin (PTx) was included as a second positive control for the overall dynamic range of the assays with most people showing antibodies against pertussis toxin at varying degrees based on earlier immunisation or infection (Table S1).

#### Specificity and cross-reactivity of the multiplex assay

To establish our bead-based multiplex assay we used two serum panels. As a negative control panel, we obtained 100 anonymised sera from healthy donors (containing 56 sera of children aged under 18 years; median age 16 years, minimal age < 1 years, maximal age 61 years) within Germany which were sent to the Robert Koch Institute between 2018/08/30 and 2019/09/27, well before the occurrence of the first cases of COVID-19 in China or Germany^1, 43^. A second panel of 524 sera contained samples from adults either positive or negative for SARS-CoV-2 IgG antibodies as determined by a commercial ELISA [Anti-SARS-CoV-2 ELISA (IgG), Euroimmun AG, Lübeck, Germany)], which were collected and analysed as part of epidemiological outbreak studies (Corona Monitoring Lokal, CoMoLo) conducted in multiple hotspots between May and September 2020 in Germany^41, 44^. The second panel was preselected to cover a range of high, medium and low positive sera as well as borderline and negative sera for validation. In total, 355 positive, 21 borderline, and 148 negative sera classified according to the ELISA were included in this panel.

**Figure 2:**
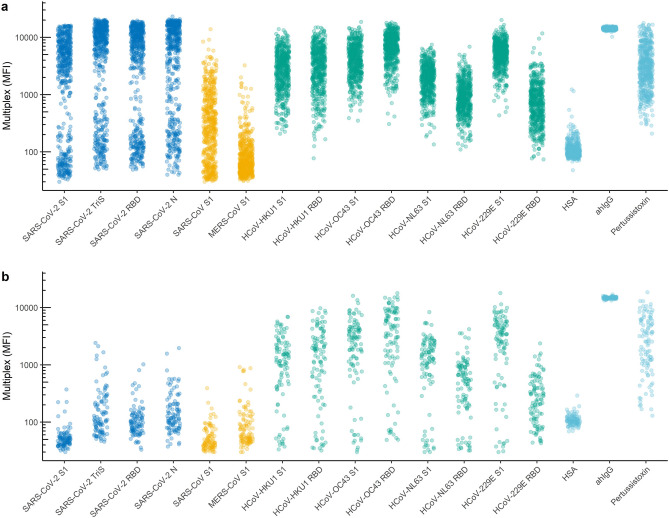
Distribution of binding signals in the multiplex assay in an outbreak panel (**a**) and a pre-pandemic panel (**b**). (**a**) Binding profile of a panel of 524 sera—sampled between May and September 2020—during epidemiological investigations of COVID-19 outbreaks in Germany against SARS-CoV-2 specific antigens (blue), SARS-CoV and MERS-CoV (yellow), and low pathogenic (green) coronaviruses HKU, OC43, NL63 and 229E, and human serum albumin (HSA), anti-human IgG (ahIgG), and pertussis toxin as controls (light blue) in the 17-plex bead-based serological suspension assay. (**b**) Binding profile of a panel of 100 pre-pandemic sera tested against the same antigens. *S1* S1 domain, *TriS* soluble ectodomain of trimeric spike protein, *RBD* receptor binding domain, *N* nucleoprotein.

When we analysed both panels with our multiplex assay, a clear bimodal distribution was detected in the outbreak panel for all four SARS-CoV-2 specific antigens (Fig. 2a, blue) whereas in the pre-pandemic panel only low binding signals occurred (Fig. 2b, blue). The low pathogenic HCoV strains HKU1, OC43, NL63, and 229E gave elevated binding signals clearly above the HSA background signals. Their signal intensities in the outbreak panel (Fig. 2a, green) showed a broad dynamic range comparable to the binding signals for pertussis toxin as expected for adults who had been in contact with many of these circulating endemic strains during their lifetime^41, 44^. The pre-pandemic panel consisted mainly of sera derived from children and young adults and showed a bimodal distribution for those who had already encountered the endemic HCoV strains (positive) and those who had not (negative). It was not possible to correlate serostatus with age in this panel as the panel was fully anonymous and randomised before measurements. We calculated the Pearson correlation coefficients between signal intensities of the sera from the epidemiological outbreak studies for the different antigens and found strong and highly significant correlations between all four SARS-CoV-2 specific antigens indicating that infection stimulates a strong immune response against both the S and N protein (Figure S2). Additionally, signals for SARS-CoV were also highly correlated with the results for SARS-CoV-2 antigens indicating significant cross-reactivity between SARS-CoV-2- and SARS-CoV-reactive sera. As expected, the results correlated highly between S1 and RBD of each HCoV strain whereas no or only very weak correlations were found between serum reactivities against spike proteins from strains SARS-CoV-2/SARS-CoV and MERS-CoV versus low-pathogenic HCoV-strains.

These results indicate that SARS-CoV-2 specific IgG antibodies can be detected without cross-reactivity against endemic HCoV strains. Significant cross-reactivity was observed for SARS-CoV, but not for MERS-CoV IgG antibodies, which was expected based on the high level of homology between SARS-CoV and SARS-CoV-2^45, 46^ whereas a broad pre-formed immunity could be described for the adult population against endemic HCoV strains.

#### Definition of reliable cut-off values for SARS-CoV-2 specific antigens by a population-based approach and a comprehensive method comparison with 7 reference assays

To minimise assay variability between individual batches of xMAP^®^ beads coupled with the same antigen, binding signals were normalised to a certified reference serum (EURM-017), which was included in every test run. Hereby, batch-to-batch variability of xMAP^®^ beads could be removed effectively lowering the inter-assay variability (see Figs. S3 and S4). To regain the initial bimodal distribution of the binding signals, normalised binding signals were log10-transformed and shifted to positive values by adding an artificial value of 3.

**Figure 3:**
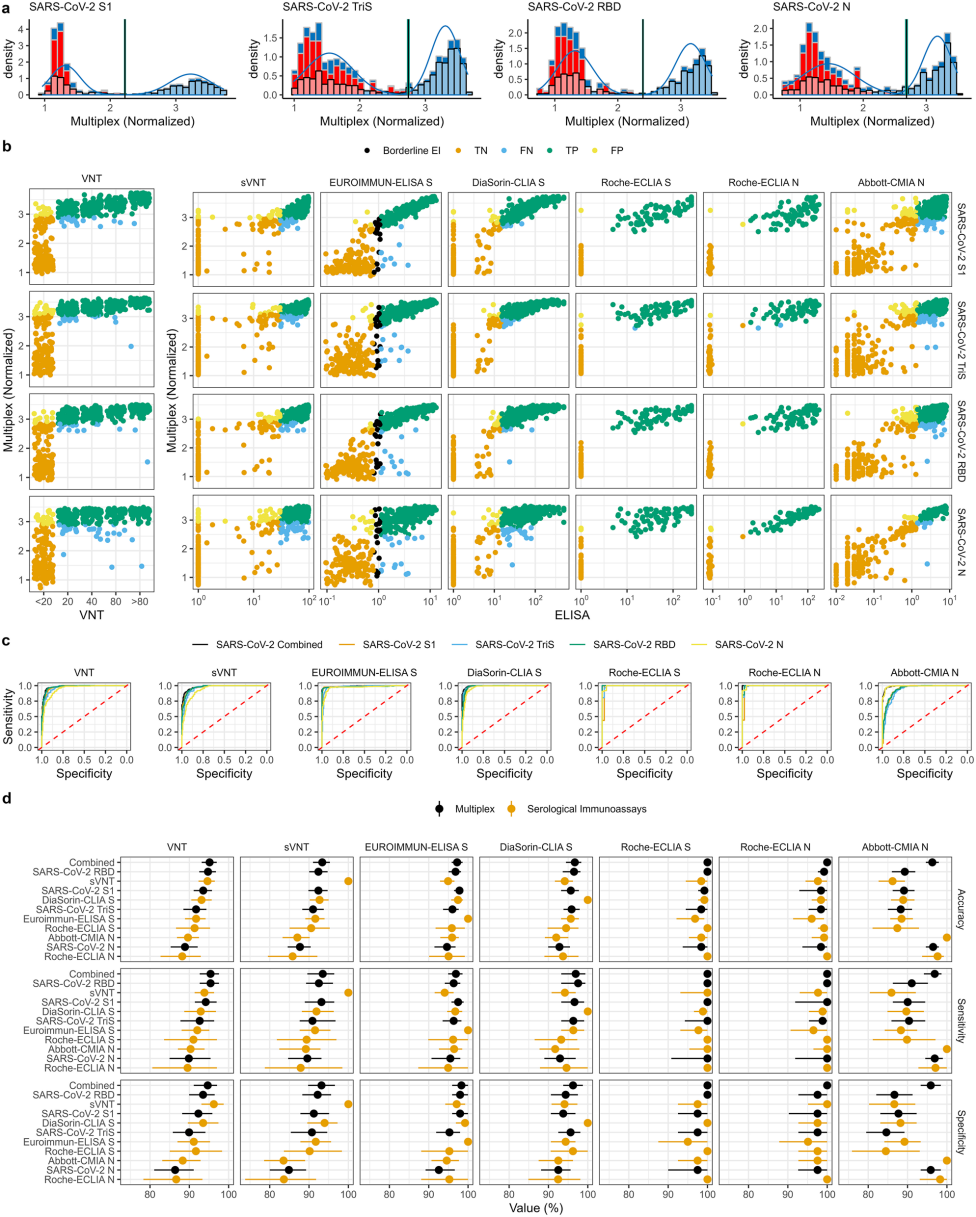
Method comparison for SARS-CoV-2 specific antigens in the multiplex assay and seven reference assays. (**a**) Histograms of normalised binding signals to SARS-CoV-2 specific antigens (S1, TriS, RBD, N antigen) measured by the multiplex assay for sera from the pre-pandemic panel (red), the outbreak panel (blue) and both panels combined (black outlines, transparent filling). Measured binding signals from the combined panels were fitted with two standard distributions (blue curves) to separate negative from positive signals in an attempt to determine a population-based cut-off (vertical lines) for each antigen. (**b**) Scatterplots with normalised multiplex assay signals for S1, TriS, RBD, and N antigens obtained by the bead-based multiplex assay established in this work (multiplex, y-axis) over results obtained for neutralising titres (VNT, cell-based neutralization assay, displayed are serum dilutions) or binding signals for the six commercial assays used for reference (sVNT, Euroimmun ELISA S, DiaSorin CLIA S, Roche ECLIA S, Roche ECLIA N, Abbott CMIA N). For each serum, measured points are colour coded according to the classification based on the cut-off values which were determined by receiver-operator-curve-analysis (ROC) (see figure c): true negative (TN, orange), false negative (FN, blue), true positive (TP, green), false positive (FP, yellow), borderline Euroimmun ELISA S (black) with each reference assay representing the respective ground truth for classification. For the comparative validation study, 486 to 512 sera were measured by the different immunoassays except for the Roche ECLIA, where a subset of 127 sera was analysed. From the overall panel, 495 sera were titrated in a cell-culture based virus neutralisation assay (VNT). (**c**) For each SARS-CoV-2 specific antigen (S1, TriS, RBD, N) ROC analyses were performed for the comparison between the results obtained by the bead-based multiplex assay in comparison to the assays mentioned in the title above the individual ROC curves. Additionally, generalised linear models were modelled from the combined responses of the four SARS-CoV-2 specific antigens and tested for the agreement with the mentioned reference methods. (**d**) Overview of the performance parameters accuracy (top panel), sensitivity (middle panel), and specificity (bottom panel) obtained from the ROC analysis for the comparison of the four SARS-CoV-2 antigens and the combined analysis of all four antigens measured with the multiplex suspension assay via a generalised linear mixed model (multiplex assay, black), and the reference assays (serological immunoassays, orange). Shown are results with upper and lower limits of 95% confidence intervals. Results are sorted by the agreement of the results from the multiplex-assay or the different reference assays with the VNT (sorted from highest accuracy to lowest).

To define cut-off values for each of the four SARS-CoV-2 specific antigens, we used two approaches: first, based on the observation that two populations of positive and negative sera became obvious in the analysed serum panel from the epidemiological outbreak studies, we used a mathematical approach by fitting two Gaussian distributions over the bimodal population density in a finite mixture model using the expectation–maximisation algorithm^47^. Population-based cut-offs with a clear separation between negative and positive populations could be calculated effectively for all four SARS-CoV-2 antigens (Fig. 3a, Table S2). Specifically, the cut-off values that best separated the two Gaussian distributions fit to the histograms of the data normalised to the certified reference serum as described above were 2.22 for the S1, 2.72 for the TriS, 2.39 for the RBD, and 2.70 for the N protein from SARS-CoV-2. Interestingly, population-based cut-off values for both the TriS and the N protein were higher as those reported for the S1 and RBD domains which could be explained by a higher level of pre-existing cross-reactive antibodies^48–50^.

Next, we compared the results of our multiplex suspension assay with results from different methods which were frequently used to detect seropositive patients: five commercial serological immunoassays, a commercial sVNT (GenScript) and a cell-based VNT assay. To this aim an extensive method comparison was performed by measuring different sample sets from the outbreak-analysis panel ranging from 127 to 512 sera in the seven different assays (Fig. 3b, Table S3). To determine the levels of neutralising antibodies, 495 sera were titrated in a cell-culture based VNT assay. Additionally, 512 sera were measured in a sVNT based on the inhibition of the RBD-ACE-2-receptor interaction by antibodies binding to the RBD in a competitive ELISA approach (GenScript). Three serological immunoassays from multiple suppliers targeting the spike protein were also included: a semiquantitative IgG-specific ELISA from Euroimmun based on immobilised S1 domain (n = 503), a chemiluminescence-immunoassay (CLIA) manufactured by DiaSorin based on immobilised full-length S protein containing both the S1 and S2 domain (n = 486) and an electro-chemiluminescence immunoassay (ECLIA) targeting the RBD supplied by Roche (n = 127). Finally, two different assays targeting the N protein were also included in the method comparison: one chemiluminescent microparticle immunoassay (CMIA) from Abbott (n = 486) and one ECLIA supplied by Roche (n = 127). Receiver-operator-curves (ROCs) were constructed for each pairwise comparison between all methods used as reference and one of the four SARS-CoV-2 specific antigens in the bead-based multiplex assay by using the results (positive or negative) from the reference methods as classifiers (Fig. 3c). Cut-off values, 95% confidence intervals, sensitivity, specificity, and accuracy were calculated using the pROC package in R (Fig. 3d)^51^. Additionally, generalised linear models comprising all four SARS-CoV-2 antigens in the bead-based multiplex assay (combined) were used for binomial logistic regression analysis for each reference method (classifier ~ S1 + RBD + TriS + N).

When we compared the results obtained by our multiplex assay with the seven assays used as reference we found a high level of agreement between the assays with slight differences depending on the antigens used (Table 1 and Table S3). For the VNT and sVNT, the highest agreement was found between the RBD and S1 domain as antigen (accuracies of 92.4 for RBD and S1 and 91 for TriS), which also mediate receptor binding, while the agreement was less pronounced for the N protein (accuracy of 87.7). Conversely, for the Abbott-CMIA—targeting the N protein—the highest agreement with our assay was found for the N protein (accuracy of 96.5) whereas less agreement was found for the S-specific antigens (89.3, 89.1, and 88.1 for RBD, S1, and TriS respectively). The overall highest agreements were found between the bead-based multiplex assay and the ECLIAs from Roche (maximum accuracy 100), but also with the ELISA from Euroimmun (maximum accuracy 97.8) and the CLIA from DiaSorin (maximum accuracy 100). As not all sera were analysed in all assays but only smaller subpanels were measured e.g. in the Roche assays, we wanted to exclude that the high level of agreement between our multiplex assay and the Roche assay was biased by sample selection. To this aim, we reran the method comparison between the bead-based multiplex assay and all reference assays based on the smallest common subpanel of 127 sera tested in the Roche assay. Here we found similar accuracies, sensitivities, and specificities indicating congruent results with the smaller subpanel (Fig. S5). When we calculated accuracy, sensitivity, and specificity for the comparison between the bead-based multiplex assay and the reference assays as well as between the different reference assays we found that our bead-based multiplex assay offered the highest accuracy compared to the other assays (Fig. 3d, Table 1 and Table S2). Exemplarily, when comparing the results obtained with the bead-based multiplex assay on all four SARS-CoV-2 antigens (combined) with the VNT-assay, 95.2% accuracy, 95.4% sensitivity and 97.2 specificity were obtained. For the other coronavirus antigens tested in our multiplex assay, only SARS-CoV S1 showed some concordance with the SARS-CoV-2 specific reference assays due to the known cross-reactivity whereas the results for MERS-CoV and the four HCoV-strains did not show any agreement with the SARS-CoV-2 specific reference methods above an accuracy of 62% indicating low cross-reactivity (Figs. S6, S7, and Table S3). As the multiplex assay addresses SARS-CoV-2 with four different specific antigens, high levels of agreement could be obtained between both neutralising and binding assays targeting either different S domains or the N protein. By combining these different antigens, the novel bead-based multiplex assay combines the advantages of assays targeting single antigens in one assay.

**Table 1:** Exemplary results for method comparisons between the cell-based virus neutralisation assay (VNT), an ELISA assay targeting the S protein (S, Euroimmun-ELISA S1) and a CMIA targeting the N protein (N, Abbott-CMIA N) with reference serological immunoassay and SARS-CoV-2-specific antigens implemented in the bead-based multiplex-assay.

Test assay/antigen	Accuracy (%)			Sensitivity (%)			Specificity (%)		
	VNT	S	N	VNT	S	N	VNT	S	N
Abbott-CMIA N	89.4	95.9	–	90.3	96.4	–	88.3	94.6	–
DiaSorin-ECLIA S	93.3	97.4	88.9	93.2	96.7	89.7	93.5	99.2	87.7
Euroimmun ELISA S1	91.7	–	88.5	92	–	88	91.1	–	89.2
sVNT	94.6	94.7	86.2	93.8	93.8	85.9	96.2	97.1	86.1
Roche-ECLIA N	88.2	95	97.6	89.6	94.9	97.1	86.7	95.2	98.3
Roche-ECLIA S	91.3	95.9	87.4	91	96.2	89.9	91.7	95.2	84.5
SARS-CoV-2 N, bead-based multiplex assay	88.9	94.6	96.5	89.9	95.5	96.9	86.4	92.6	95.9
SARS-CoV-2 RBD, bead-based multiplex assay	94.7	96.8	89.3	95.4	96.3	91.1	93.5	98.0	86.7
SARS-CoV-2 S1, bead-based multiplex assay	93.5	97.8	89.1	94.5	97.5	90	92.3	98.0	87.7
SARS-CoV-2 TriS bead-based multiplex assay	91.7	96	88.1	92.6	96.6	90.4	90.5	95.3	84.6
SARS-CoV-2 combined, bead-based multiplex assay	95.2	97.2	96.3	95.4	96.9	96.9	97.2	98.6	95.9

For full results see supporting Table S3.

Finally, to determine a reliable and robust cut-off value for each of the four SARS-CoV-2-specific antigens in our bead-based multiplex assay, we used the cut-off values determined by the ROC-analysis and calculated a median cut-off for either S- or N-specific assays by using a selection of specific values based on the following assumptions: We did not include the cut-off values for the VNT or the sVNT since within those assays only neutralising or receptor-binding blocking antibodies are detected. Hence binding, but non-neutralising antibodies are not detected^29^. In consequence, the determined cut-off values would overestimate the binding assays, which more closely mirror the assay principle of our bead-based multiplex assay detecting all binding antibodies. Consequently, for the S-specific antigens (S1, RBD, TriS), the median cut-off was calculated from the population-based cut-off and the three S-specific ELISA assays (Euroimmun, DiaSorin, Roche). For the N-specific cut-off the median cut-off was calculated from the population-based cut-off and the cut-off from the Roche N-ELISA while the Abbott-ELISA was omitted since the cut-off used for initial classification as supplied by the manufacturer was too high (see Table S3 and Fig. S8). Based on these calculations, the following normalized cut-off values were determined for the SARS-CoV-2-specific antigens in our bead-based multiplex assay to discern positive from negative sera: for TriS 2.76 (95% confidence interval (CI) ranging from 2.70 to 2.90), for S1 2.48 (2.42–2.53), for RBD 2.51 (2.46–2.57), and for N 2.59 (2.52–2.72), respectively.

To see how seroprevalences based on these cut-off values would compare to the numbers determined by a reference assay, we calculated the number of positive and negative sera in both panels used for the assay establishment (outbreak panel n = 524, pre-pandemic panel n = 100) and compared those results with the number of positive and negative sera that were determined by a commercial ELISA targeting the S protein S1 (Euroimmun) (Table 2). For the outbreak-panel we found highly comparable results of approximately 70% positive sera determined by both the commercial ELISA and the four SARS-CoV-2 specific antigens. The commercial ELISA classified 21 sera as borderline, which had been excluded from the calculation of the positive rate. The level of ambiguity was comparable when the upper and lower limits of the 95% CI of the SARS-CoV-2 specific antigens included in the multiplex assay were considered for the TriS (18 sera) or N protein (23 sera), but much smaller for the S1 domain (4 sera) or RBD (2 sera). However, in the pre-pandemic panel, 4 sera, although with overall low signal intensities, were classified as positive by the reference Euroimmun ELISA whereas only 1 serum was classified as positive by one of the four antigens (TriS) in the multiplex assay. This indicates a higher specificity of our multiplex assay with a clearer separation between positive and negative samples at comparable sensitivity.

**Table 2:** Comparison between number of positive and negative sera (95% confidence intervals in round brackets) in the outbreak or pre-pandemic panels used for assay establishment as determined by the new bead-based multiplex assay for each antigen or a commercial ELISA targeting the S protein (Euroimmun-ELISA S1).

Antigen/assay	Outbreak panel				Pre-pandemic panel		
	Positive	Negative	BL^a^	Positive (%)	Positive	Negative	BL
S1	372 (369–373)	152 (151–155)	–	71.0	0 (0)	100 (100)	–
TriS	365 (351–369)	159 (155–173)	–	69.6	1 (0–1)	99 (99–100)	–
RBD	363 (361–364)	161 (160–163)	–	69.1	0 (0)	100 (100)	–
N	355 (338–361)	169 (163–186)	–	67.7	0 (0)	100 (100)	–
Euroimmun	355	148	21	70.6	4	96	0

^a^Borderline (BL) results as defined by the supplier.

In conclusion, our results indicate that the bead-based multiplex assay enables a highly sensitive and specific detection of SARS-CoV-2 specific antibodies by addressing multiple targets on the S protein as well as the N protein.

### Modular setup and robust assay performance make the bead-based multiplex assay a versatile tool for different applications

#### Highly correlated detection from serum and dried blood spots further broadens the applicability of the assay

To further expand the field of potential applications for our bead-based multiplex assay, we compared the detection of IgG from serum with a detection from DBS material for a panel of 86 sera. Paired samples, where both serum and DBS were collected, were obtained within the scope of the hotspot studies in Bad Feilnbach^41^ and measured by both commercial ELISAs (N- and S-specific, Euroimmun) and our bead-based multiplex assay performed with reconstituted DBS in comparison to paired serum. We calculated linear regressions between the results obtained from serum and DBS for all multiplex antigens as well as the two commercial ELISAs used to analyse the panel (Fig. S9). Here, we found an overall excellent accordance between the results obtained from reconstituted DBS and serum for all HCoV- and SARS-CoV-2 specific antigens (R^2^ values from linear regressions of z-score normalised results > 0.94). The only exception was SARS-CoV S1 and, to a lesser degree, MERS-CoV S1 in the multiplex assay, where seemingly lower R^2^ values were obtained (R^2^ values of 0.55 and 0.65 respectively). For those analytes, a better comparison of serum versus DBS would require the analysis of samples from SARS-CoV and MERS-CoV positive individuals. However, reliable results for all specific antigens could be obtained by the bead-based multiplex assay from both serum and DBS indicating that the bead-based multiplex assay is well suited for antibody detection from DBS (Fig. S9b). This result is important especially for the planning and execution of large population-based screening programmes since collection and storage of DBS offers several advantages over the use of serum, e.g., as minimally invasive method it requires simple handling steps only, that can be performed at home without medical personnel. Successful sampling has already been shown within a nationwide seroprevalence study for anti-SARS-CoV-2 antibodies in Germany^52^. Additionally, DBS show a high sample stability (for the analysis of antibodies and many other analytes in the blood) and storage at ambient temperature is usually sufficient over an extended period of time.

#### Association between seroprevalence against SARS-CoV-2 and the four endemic HCoV-strains during outbreaks in day-care centres

**Figure 4:**
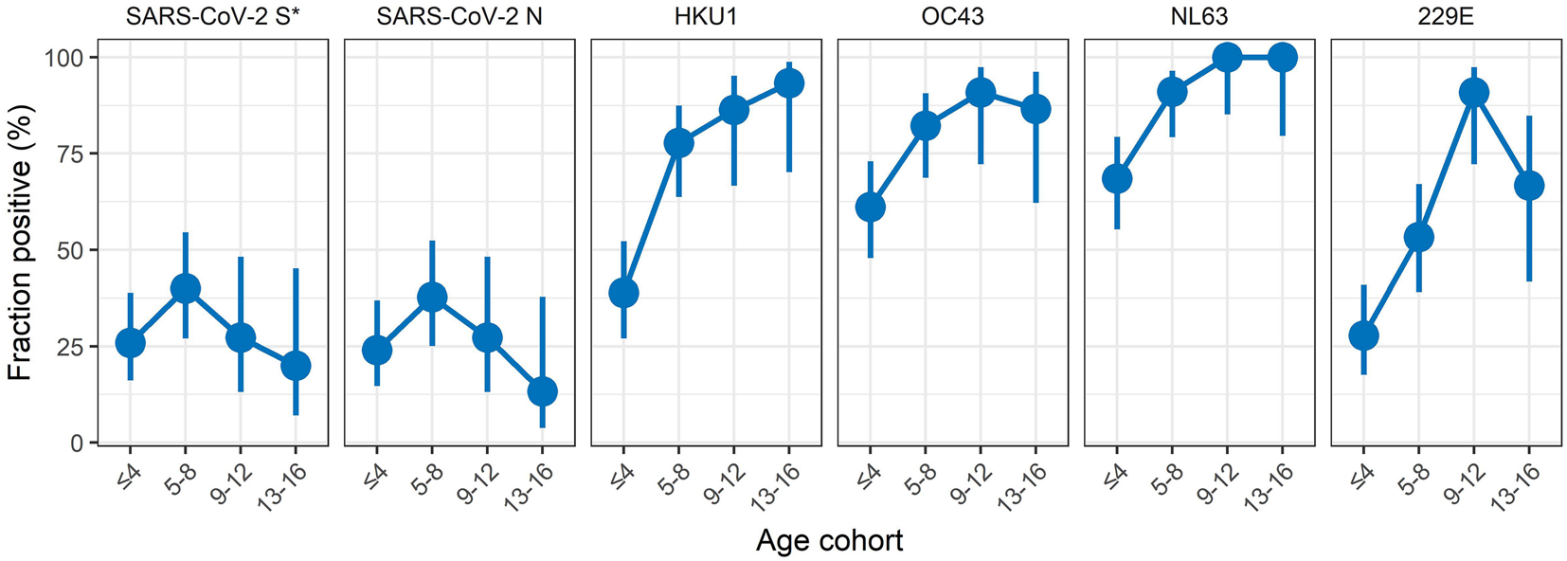
Seroprevalence against SARS-CoV-2 and endemic HCoV-strains in a serum panel of children. Seroprevalence in a cohort of four age cohorts (age in years) was analysed using the population-based cut-off value for the four HCoV-strains (Fig. S10) or the established robust cut-off for SARS-CoV-2 S and N antigens (Fig. 3a and Table S2). The proportion of positive sera is shown in the respective age cohort with calculated 95% confidence intervals^57^. Asterisk: for the S-specific antigens (TriS, S1, RBD), a combined serostatus was determined as described in the supporting information.

To demonstrate the versatility of our assay, we analysed a panel of n = 137 sera collected from children aged 1 to 16 years in the scope of SARS-CoV-2 outbreaks in day-care centres in Berlin from 10/2020 to 06/2021^53^. Day-care centres with at least one recent laboratory-confirmed SARS-CoV-2 case were included in the study. DBS were collected from both children and close household members and the serostatus was determined using an S-specific commercial ELISA (Euroimmun). Our main interest was to test whether our assay allowed us to measure the seroprevalence against SARS-CoV-2 and the four endemic HCoV strains simultaneously. Since within an adult population almost all sera are seropositive for the endemic HCoV strains, a panel of young children was chosen to determine an increase of seropositivity with increasing age. Apart from that, we were interested in a possible correlation between the immunity to endemic HCoV strains and SARS-CoV-2. It was shown that a cross-reactive immune response against endemic HCoV strains exists due to cross-reactive antibodies^54^, or a cross-reactive T-cell response^55^. The antigens used in our assay to distinguish between the HCoV-strains and SARS-CoV-2 are the highly-specific domains containing the RBD and S1 domains and not the S2 domain and the N protein, which share a higher level of homology between SARS-CoV-2 and the other HCoV strains^10^. Hence, we would not expect to pick up cross-reactive antibodies, but instead measure more broadly how a specific serostatus (positive or negative) against one of the four endemic HCoV strains might impact the serostatus against SARS-CoV-2. To this aim, we determined the SARS-CoV-2 specific serostatus using the cut-offs defined as determined in Fig. 3 a and Table S2. For a combined S-specific serostatus, results for all three S-specific antigens (TriS, RBD, S1) were combined using an algorithm as described in the supporting information. To define cut-off values for the HCoV-specific antigens we used the pre-pandemic panel (n = 100) containing both negative and positive sera to determine a population-based cut-off as described above (Figure S10). Hereby, we were able to discern positive from negative sera in the sample population and to follow the seropositivity in dependence of age (Fig. 4). In agreement with the literature, we found increasing seropositivity with increasing age for all four HCoV strains analysed. Conversely, seropositivity for SARS-CoV-2 was independent of age. This agrees well with the difference between endemic viruses like the four HCoV strains, which infect children at a young age leading to accumulating immunity with increasing age^56^ and the pandemic outbreak of SARS-CoV-2 which affected all age groups equally within a short time frame. When we calculated the odds ratios for SARS-CoV-2 seroconversion in dependence of the serostatus against the four endemic HCoV strains we did not find a statistically significant impact of the HCoV-serostatus on the SARS-CoV-2 specific serostatus (odds ratio (OR) SARS-CoV-2 S and 229E: 1.1; HKU1: 0.9; NL63: 1.2; OC43: 1.15). This indicates that previous infections with endemic HCoV strains did not protect from SARS-CoV-2 infection in our study panel. Nevertheless, our data show that by defining a population-based cut-off for the endemic HCoV coronaviruses, our assay enables serological studies for both SARS-CoV-2 and the four endemic HCoV strains simultaneously from minimal sample volumes.

#### Analysis of time course and IgG-subclass specific immune response in hospitalised COVID-19 patients in dependence of disease severity

**Figure 5:**
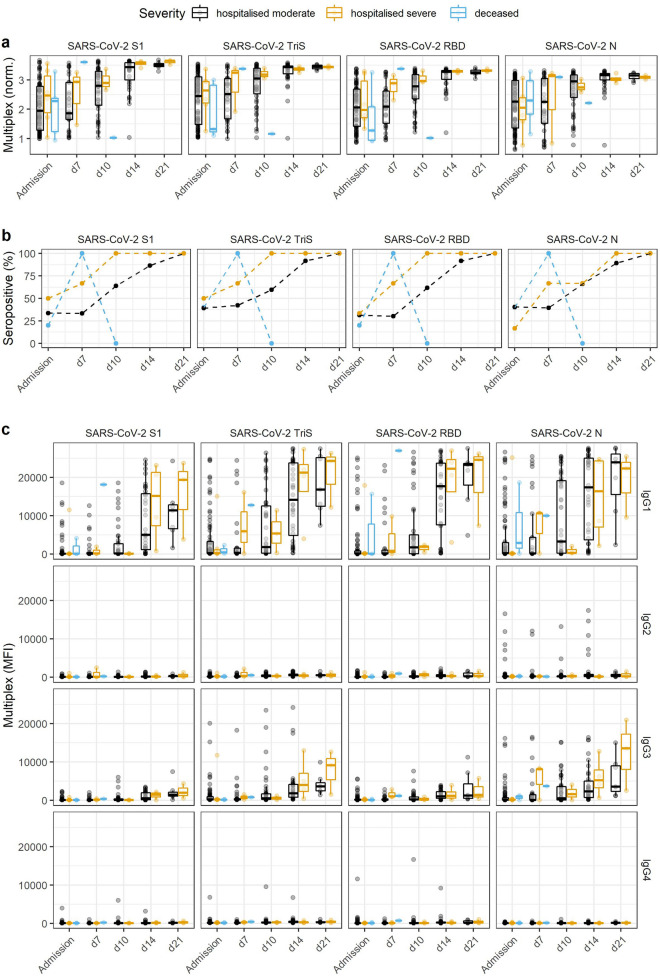
Time course of IgG response in patients hospitalised with moderate or severe symptoms or who deceased, as determined by SARS-CoV-2 specific antigens in the bead-based multiplex assay. (**a**) Normalised binding signals of patients with mild or severe clinical symptoms during hospital admission or who deceased during the study; blood was drawn upon admission and up to 21 days after symptom onset. Initially, 100 patients were sampled at the day of admission, 41 patients could be followed until day 14 after symptom onset and 10 patients until day 21 after symptom onset. (**b**) By using the previously established cut-off values, the fractions of seropositive sera in both panels were calculated over time. (**c**) Using IgG-subclass specific antibodies, the relative contribution of the four IgG-subclasses to the SARS-CoV-2-specific antibody immune response was analysed over time in both patient panels. Results for HCoV-antigens are shown in Fig. S11.

To test our bead-based multiplex assay in a clinical setting, we analysed the time course of the IgG antibody immune response in a panel of sera from hospitalised patients with COVID-19. Based on clinical symptoms and outcome, patients were classified as hospitalised with moderate or severe symptoms, or deceased. Sera were taken at hospital admission and 7, 10, 14 or 21 days after symptom onset (provided the patients were hospitalised on these dates) to monitor the development of the IgG-response and seroconversion rates by using our bead-based multiplex assay (Fig. 5a, b and Table 3). While initially 110 patients were included in this study, we were able to follow 41 patients until day 14 after symptom onset and ten patients until day 21 after symptom onset. We found that initially approximately one third of the patients had already developed antibodies against SARS-CoV-2 when administered to the hospital with no differences between patient groups classified by severity or outcome. Following the time course, we found that patients who developed severe symptoms seroconverted earlier and complete until 14 days after symptom onset while patients with only moderate symptoms showed a slightly lower conversion rate with approximately 90% seroconversion until day 14. Results for patients who deceased were inconclusive due to the small number of patients that could be followed. Additionally, we analysed the contribution of different IgG-subclasses to the IgG immune response in our bead-based multiplex assay. To this aim, serum antibodies bound to the four SARS-CoV-2-specific antigens were detected by either IgG1-, IgG2-, IgG3-, or IgG4-specific detection antibodies. Here we found that the antibody response was dominated by IgG1, followed by IgG3. IgG2 or IgG4 antibodies were only detected in sera of single patients by our assay (Fig. 5c). For the four HCoV-strains no clear trend was seen (Fig. S11).

**Table 3:** Time course of seroconversion in a panel of hospitalised patients depending on the severity of symptoms and clinical outcome.

Timepoint	Severity	Sera (n)	S1 positive (%)	TriS positive (%)	RBD positive (%)	N positive (%)
Admission	Hospitalised moderate	89	34	39	31	40
	Hospitalised severe	6	50	50	33	17
	Deceased	5	20	40	20	40
d7 after symptom onset	Hospitalised moderate	33	33	42	30	39
	Hospitalised severe	3	67	67	67	67
	Deceased	1	100	100	100	100
d10 after symptom onset	Hospitalised moderate	47	64	60	62	66
	Hospitalised severe	3	100	100	100	67
	Deceased	1	0	0	0	0
d14 after symptom onset	Hospitalised moderate	37	86	92	92	89
	Hospitalised severe	4	100	100	100	100
	Deceased	0	–	–	–	–
d21 after symptom onset	Hospitalised moderate	7	100	100	100	100
	Hospitalised severe	3	100	100	100	100
	Deceased	0	–	–	–	–

#### Antibody binding profiles allow a differentiation between immunised and infected patients

Next, we used our bead-based multiplex assay to analyse the immune response both after immunisation and infection. To this aim, panels of up to 20 sera comprised of patients who were either immunised by the Astra Zeneca vaccine (Vaxcevria^®^), the BioNTech vaccine (Comirnaty^®^), or which were infected by either the Alpha (B.1.1.7) or Beta (B.1.351) variant were analysed with our bead-based multiplex assay (Figs. S11, S12 and Table S4). As expected, all patients immunised with both vaccines reacted strongly with the S antigens. Three patients, who were already infected before the first vaccination showed 100% seroconversion on the S antigens and 2 out of those 3 sera were also rated positive on the N antigen (Table S4). When all 20 sera were analysed, between 80 and 90% of the sera tested positive on the S antigens after immunisation with the Astra Zeneca vaccine after the first immunisation, depending on the antigen, while between 95 and 100% tested positive after one immunisation with the BioNTech vaccine. Data after the second vaccination were only available for the subjects immunised with the BioNTech vaccine. Here, a clear increase of the overall binding signals was seen (*t*-test p ≤ 0.0001, Fig. S12a) with complete seroconversion for all 20 sera tested. With regard to the N protein, two sera from the patients who had been immunised with the Astra Zeneca vaccine tested positive while all other sera tested negative. As patients with known previous infections were excluded from this analysis, these results indicate unrecognised infections between the first vaccination and serum sampling 6 weeks after. When we compared the immune response after infection with either Alpha or Beta SARS-CoV-2 variants we found that for the Alpha variant approximately 90% seroconverted after infection while for the Beta variant differences were seen with regard to the different antigens (Fig. S12b, Table S4). While 100% of all 16 sera tested positive on the N protein as antigen, 87.5% tested positive on the TriS protein, 75% tested positive on the S1 domain, and only 62.5% tested positive on the RBD. This could be explained by lower binding of Beta-specific sera to the S1 and RBD antigens derived from the wildtype Wuhan-Hu-1 sequence implemented into our assay^58^. For comparison, no significant difference in binding to HCoV-specific antigens was seen either after immunisation or infection (Fig. S13). This data shows that reactivity against the N protein can be used as a marker for previous infection independent of vaccinations against the S protein.

#### Determination of IgM/IgG ratios enables discrimination of recent from previous infections

After showing that IgG reactivity against the N protein can be used to discern infected from immunised individuals, we wanted to expand our assay further to discriminate sera that have been sampled early after onset of infection from sera that have been collected at later time points. The idea was to use such an assay to test, if a current PCR confirmed infection might have been preceded by an unknown or undiagnosed SARS-CoV-2 infection. In that case, the immune response would be dominated by IgG antibodies while for primary infections, IgM would be expected at early time points. Identification of previous infection is important for studies on vaccine efficacy since exclusion of patients with an infection history is often considered in case–control studies. On the one hand, COVID-19 patients classified as cases in these studies have a lower likelihood to be vaccinated if previously infected (vaccination is usually not recommended shortly after infection), and on the other hand, antibodies from previous infections may confer partial protection. Both factors can potentially bias study results and make identification and exclusion of such patients necessary. Our sample panel consisted of 54 paired samples from 27 patients with PCR-confirmed SARS-CoV-2 infections, mainly infected with the Delta or Omicron variants based on the time points of infection ranging between 2021/08/09 and 2022/02/23. The panel contained patients hospitalised for severe SARS-CoV-2 infections with or without previous vaccination history from a study to monitor vaccine efficacy^59, 60^. Serum was collected at two time points: either from 0 to 6 days after onset of symptoms (early time point), or at least 17 days up to 99 days after onset of symptoms (late time points). By comparing the antibody immune response between the early and late time points we also wanted to test how well the immune response against the N protein was suited to discover breakthrough infections after immunisation. High seroconversion rates at late time points would indicate that the immune response against the N protein is reliably triggered, even after previous immunisation with S-specific vaccines, at least in our panel of patients.

To this aim we analysed all samples with our bead-based multiplex assay for both the IgG- and IgM-antibody response against the four SARS-CoV-2-specific antigens. Detection of bound antibodies was achieved with a µ-chain specific anti-IgM antibody instead of the Fc-γ-specific IgG antibody used otherwise. The assay was validated against one commercial RBD- and one N-specific ELISA with satisfying sensitivities between 84.3% (S1) and 90.6% (RBD) at specificities between 78.2% (RBD) and 89.1% (S1) for the S-specific assays and 84.1% sensitivity at 91.7% specificity for the N-specific assays (Fig. S14).

**Figure 6:**
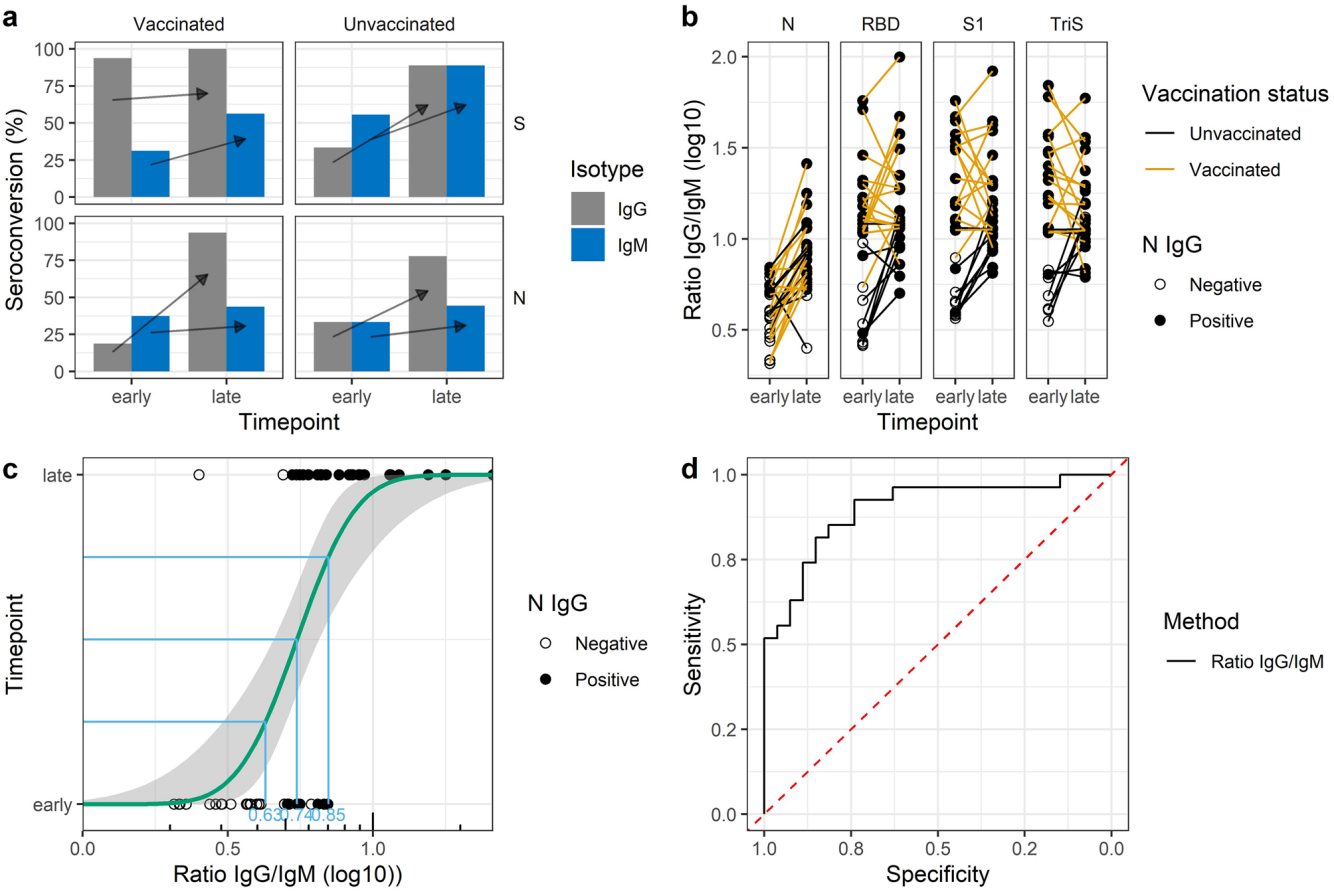
Discrimination of recent from previous SARS-CoV-2 infections using the IgG/IgM-ratio. (**a**) Seroconversion rate in previously vaccinated or unvaccinated patients on the S or N antigen for the IgG or IgM immune response at early (< 7 days after symptom onset) or late (> 17 days after symptom onset) time points. (**b**) Change of IgG/IgM ratios in paired samples between early or late time points on the N, RBD, S1, and TriS antigens, stratified by SARS-CoV-2 vaccination status and serostatus with regards to the IgG response against the N antigen in the multiplex assay (**c**) Probit analysis to discern sera sampled at early or late time points based on the IgG/IgM ratio on the N antigen. (**d**) ROC for the results of the probit analysis shown in c.

When we analysed the rate of seroconversion in the panel of 25 patients (two patients were omitted from this analysis due to missing information with respect to their vaccination status), we found marked differences between the IgG and IgM response for the S and N antigens depending on the time point after symptom onset (Fig. 6a). As expected, in vaccinated patients IgG seropositivity on the S antigens was already high at the early time point after symptom onset and increased to 100% at the late time point. Conversely, only approximately one third of unvaccinated patients showed seroconversion with IgG antibodies at the early time point, which increased to almost 90% at the late time point. On the N antigen, IgG seropositivity rates in the vaccinated patients were slightly lower at the early time point (19 vs. 33%), but increased higher at the late time point compared to the unvaccinated patients (94 vs. 76%). With regard to the IgM response on the S antigen in vaccinated patients, seroconversion rose from 31% at the early time point to 56% at the late time point whereas in unvaccinated patients a much stronger IgM response was induced starting at 56% at the early time point and rising to 89% at the late time point. On the N antigen, the immune response between vaccinated und unvaccinated patients was similar with about 35% IgM seropositive patients at the early time point and about 44% IgM seropositive patients at the late time point. These results indicate that the IgM response was much stronger on the S antigens compared to the N antigen in the unvaccinated group. Additionally, differentiation between early and late infections based on the IgG and IgM seroconversion rates on the N antigen was not sufficiently clear as IgM and IgG positive sera appeared both at the early and late time points. However, we were encouraged by these findings that the ratio between IgG and IgM seropositivity was high at the late time points for the N protein with a larger proportion of patients having seroconverted for IgG compared to IgM. Conversely, the ratio of the IgG seroconversion rate to the IgM seroconversion rate was already high at the early time point on the S protein in the vaccinated patients, and stayed high at the late time points.

Hence, we investigated if the IgG/IgM-ratio would be a better classifier for early and late time points. When we compared the IgG/IgM-ratios between the early and late time points for the paired serum samples we could observe a clear increase in the ratios in almost all paired samples on the N antigen irrespective of the vaccination status congruent with an IgG dominated immune response at the later time points (paired *t*-test p < 0.0001, Fig. 6b). Similar results were seen for the S antigens, but only in unvaccinated patients (paired *t*-test p = 0. 0.0016 for the S1 for the difference between early and late time point IgG/IgM-ratio), while the ratios were already higher at early time points in vaccinated patients (paired *t*-test p = 0.57 for the S1 antigen). These results indicate that individual IgG/IgM-ratios in patients can hint towards the time point after infection and hence, if a previously undiagnosed infection might have occurred.

Finally, we performed probit analysis with early and late time points as possible binomial response variables over the log10-transformed IgG/IgM-ratio to define a cut-off to discriminate early from late responses on the N antigen (Fig. 6c). We used this cut-off as input for a ROC analysis to test the ability of this method to correctly classify the sera into early or late time point (Fig. 6d). Here, we found an area under the curve (AUC) of 0.83 indicating the method was able to classify 45 out of all 54 analysed sera correctly. This shows that based on IgG/IgM-ratios, a classification of sera in early or late time points is possible in most cases. Hence, high IgG/IgM-ratios on the N antigen at early time points after symptom onset of an acute SARS-CoV-2 infection are strong indicators of previous infections.

## Discussion

Serological assays are pivotal for our understanding of SARS-CoV-2 epidemiology, induction of antibody immune responses after infection or immunisation as well as possible links to disease severity or outcome of COVID-19 in patients^19, 22–24^. Some questions can be answered exclusively by serological assays including the estimation of underreporting infections in the general population^61, 62^. Hence, a multitude of assays, both commercial and in-house, have been developed since the outbreak of SARS-CoV-2 in 2019. During that time, various research questions and consequently different requirements were relevant for serological assays and those requirements are ever changing. While speed was of utmost importance during the initial phase of development for serological assays^10, 11^, high specificity was also needed to ensure that accurate results could be obtained in population-wide studies or studies in specific demographic subpopulations^63^ despite potential cross reactivity with endemic HCoV strains^22, 48, 62, 64, 65^. Hence, selection of highly specific antigens, ideally in conjunction with a test for specificity by also including endemic HCoV-strains simultaneously was crucial^66, 67^. Besides this, the overall kinetics and persistence of the immune response^68–70^ and antibody class switching was deciphered using serological assays by measuring the IgA, IgM, and IgG responses^71, 72^. With the introduction of the first vaccines, research focused on the induction and endurance of protective immunity as well as the search for correlates of protection^18, 73^. However, with the ever-increasing number of different serological methods, research also focused on comparisons to identify the most specific and sensitive assays^7, 74, 75^ as well as to harmonise results obtained by different methods^76^.

In this work we present data on the validation and implementation of a 17-plex bead-based multiplex assay in multiple research settings. The assay was thoroughly validated against six commercial assays and the “gold standard”, the VNT, performed in the consultant laboratory for coronaviruses in Germany. Due to its excellent performance with regards to sensitivity and specificity the assay has, besides the examples shown here, already been used in epidemiological settings as well as clinical studies^59, 77, 78^. Despite the large number of publications describing serological assays for SARS-CoV-2, we believe that our work can still contribute to the body of scientific literature for several reasons. First, by covering all human pathogenic coronaviruses, our bead-based multiplex assay is among the most complete and comprehensive assays published. For assays based on the same technical platform, the number of antigens addressed ranged between 3^79–81^, 5^40^, 8^68^, 12^82^, and up to 18 antigens^83^. Although even higher numbers of multiplexing have been reached by targeting the complete proteome of SARS-CoV-2 in addition to other proteins^84^, the number of relevant antigens with high immunogenicity was limited to S- or N-derived antigens of SARS-CoV-2, SARS-CoV, MERS, or the four endemic HCoV-strains.

Here, with the comparison to six commercial assays targeting both the S or N antigen from SARS-CoV-2, our work also represents one of the most extensive studies describing a newly developed method. Although not all assays could be performed with the complete set of sera used for validation (up to 624 sera) due to technical reasons, we were able to show that results for assay sensitivity and specificity were not skewed by the selection of different subpanels. Even the smallest number of sera used for the method comparison with 127 sera was still in range of other studies developing new assays for the parameters. Overall, the agreement between the different assays as well as the agreement between our multiplex assay and the different commercial assays as well as the neutralisation assay was generally high, similar to other studies that performed systematic method comparisons between commercial assays and/or neutralisation tests^33, 85–88^. One limitation of our study is that the validation was performed solely against SARS-CoV-2 specific reference assays. That means that the performance of antibody detection against the other coronaviruses still needs to be validated independently.

The highest level of agreement was reached comparing assays addressing similar antigens or functionalities. This means that assays targeting the N protein correlated best with other assays addressing the N protein, or that the VNT assay correlated best with the sVNT or other assays targeting the RBD, while S-specific assays showed the highest correlation with other S-specific assays^89^. This has two important implications: First, by including multiple SARS-CoV-2-specific antigens targeting domains on the S protein as well as the N protein, we were able to address neutralising antibodies by including the RBD, which correlates most with the results of VNT and sVNT assays, and simultaneously cover N-specific assays by including the N protein as antigen. Thereby we could also show that including both antigens is crucial for differentiation between infection and immunisation, although seroconversion rates on the N protein in previously vaccinated patients have been shown to be lower compared to seroconversion in naïve patients after infection^90^. In our study we report higher seroconversion rates in our small panel of vaccinated patients compared to unvaccinated patients. This could, however, be skewed by the small sample size as well as the focus on patients hospitalised due to COVID-19. Second, to find reliable cut-off values for our newly established assay, antigen-specific reference methods targeting the same proteins/domains as covered in the bead-based multiplex assay had to be included. Otherwise, suboptimal or skewed cut-off values might have a negative impact on the overall assay performance. We averted this by calculating individual antigen-specific cut-off values by inclusion of only matching reference methods and excluding methods targeting other antigens or measuring virus neutralisation, as this would have led to overestimated cut-off values. Interestingly, we found that the cut-off value for the N-specific assay by Abbott supplied by the manufacturer was set rather high. This might explain the observation in a longitudinal study that the initially high sensitivity of the Abbott assay declined more rapidly compared to other assays in the given study^18^. Additionally, we could show that with our set of sera used for the validation in a naïve population, cut-off determination by fitting density distributions in the absence of a reference assay was also possible. Overall, we believe that the determined cut-off values for the different bead sets or antigens, respectively, are highly reliable given that they are based not only on one but several method comparisons combined.

Another advantage of our assay is the normalisation to the EURM-017 standard serum^91^. Besides minimising inter-assay variability, conversion into BAU/mL (Binding Antibody Units) as expressed by the WHO international standard 20/136 could be achieved, although the focus of this work was to determine a reliable cut-off to discern positive from negative sera. However, quantitative results with high correlation to neutralisation and protection are becoming more and more important the more people were infected; therefore, this issue will be addressed in future work. Of great technical value for future studies is the fact, that our bead-based multiplex assay, as others but clearly not all assays, works equally well from serum and dried blood spots^92–103^. Hereby, easier and broader sampling, shipment, and storage enables large epidemiological studies without compromising the quality of the results.

The value of our assay was finally confirmed by the congruent results we obtained compared to data from the literature with regards to the tested applications. Congruent with other publications, we could show that a more severe disease progression is accompanied by a stronger and earlier IgG immune response^81, 89, 104–108^. We could also confirm that the antibody response is dominated by IgG1 and IgG3 subclasses^39, 109^ and that both infection and vaccination induce a strong immune response on the S and N or, for the latter, only on the S protein, respectively^15, 24^. We further analysed the impact of pre-existing immunity against the four endemic HCoV-strains on the rate of seropositivity against SARS-CoV-2 in a small panel collected during outbreaks in day-care centres in Germany. Here, we did not find a significant impact of previous HCoV infections on subsequent SARS-CoV-2 infections. However, the study was not designed to answer this question explicitly but was more a proof-of-principle to show that our assay was able to measure seroprevalences against HCoV-strains and SARS-CoV-2 simultaneously. This was made possible by developing a population-based cut-off for the four low-pathogenic HCoV-strains, making our assay also useful for studies targeting endemic coronaviruses^83^. Hereby we could determine the seroprevalence in a pre-pandemic panel of children and young adults for all four HCoV-strains, confirming the overall dynamics and kinetics described in literature^56^. Finally, it is important to note that our assay was not designed to evaluate the extent of cross-reactive antibodies between endemic HCoV strains and SARS-CoV-2, as we have included the highly specific S1 and RBD domains in our assay and not the more conserved S-trimers or N proteins of the endemic HCoV strains^110^. It was shown that such antibodies exist and that they are boosted upon infection with SARS-CoV-2, but that they are not associated with protection^111^.

Finally, a novel aspect of our work is the use of the IgG to IgM ratio on the N protein to differentiate in retrospect early time points from late time points after infection. Since the antibody immune response against SARS-CoV-2 is characterised by an early onset of the IgG response only shortly after the IgM response^112, 113^, determination of IgG or IgM alone can be inconclusive with regard to the time after symptom onset. However, IgG antibodies are longer-lasting compared to IgM antibodies^114–116^, hence an increased IgG to IgM ratio is expected the more time passed since symptom onset. We have decided to measure the IgM response as a marker of early infection based on our previous experience with setting up IgM-specific multiplex-assays^117^. However, due to the strong and early induction of an IgA response after SARS-CoV-2 infections, IgA would also have been a feasible alternative to discern the early from the late immune response^118^. Results might even improve given the fact that the IgA response is stronger as compared to the IgM response^119, 120^. However, as the IgA response is also more persistent, it might be more difficult to discern early from late timepoints^121^. The discrimination based on the IgG to IgM ratio could be especially useful in unvaccinated patients undergoing an acute PCR-diagnosed and symptomatic infection to test if a previous, but possibly unrecognised or undiagnosed infection could offer some protection against the current infection. Additionally, such information has to be taken into consideration in studies determining vaccine efficacy since a previous infection also offers protection against severe disease^122, 123^ depending on the time since infection, and unrecognized previous infections might confuse results, especially since the emergence of the Omicron variants^124, 125^.

In conclusion, our bead-based multiplex assay is a valuable tool for SARS-CoV-2 related research questions. Excellent sensitivity and specificity, discrimination between vaccinated and infected patients based on N protein reactivity, the ability to detect both IgG (including subclass-specific antibodies) and IgM and its implementation to discern recent from previous infections ensure broad applicability to comprehensive epidemiological studies. A highly reliable and robust cut-off was defined by a thorough and comprehensive method comparison including six commercial assays targeting anti-S, anti-N, and neutralising antibodies in addition to a comparison to the “gold-standard” VNT assay. Additionally, a robust population-based cut-off based on signal-distribution enables unbiased discrimination of positive from negative sera for both SARS-CoV-2 specific antigens as well as HCoV-specific antigens, further broadening the scope of applications for our assay. Another strongpoint is the flexible adaptability of the bead-based multiplex assay to ever-changing requirements due to manifold research questions as exemplified by studies linking clinical outcome to IgG immune responses, analysis of IgG subclass usage, or questions regarding the duration since the last infection by implementing different isotype or subclass-specific detection antibodies. Future work will target the immune response against different variants of concern (VOCs) by expanding our panel of SARS-CoV-2 specific antigens with RBD and/or S1 domains of important VOCs like Delta or Omicron^126, 127^ to monitor population-wide antibody immune responses in seroepidemiological studies.

## Methods

### Serum panels and ethics statements

The following serum panels were used for the establishment and validation of the assay: a total of 100 sera, which have been collected between August 2018 and September 2019 for routine testing against measles, mumps, and rubella IgM and IgG antibodies were anonymised and used as a pre-pandemic panel before the emergence of SARS-CoV-2. The panel was comprised from 41 sera from patients aged 10 years or younger, 17 sera from patients aged between 11 and 20 years, 20 sera from patients aged between 21 and 30 years, 6 sera from patients aged between 31 and 40 years, 7 sera from patients aged between 41 and 50 years, and 9 sera from patients aged 51 years and older. The serum status was determined using a commercial ELISA to quantify IgG antibody binding to the SARS-CoV-2 S1 domain (Anti-SARS-CoV-2 ELISA (IgG) Euroimmun, Lübeck, Germany). A panel comprising 524 sera, which were collected between May and June 2020 during early hotspot studies performed in Germany containing both positive and negative sera, was used as the main serum panel for validation of the assay by method comparison^41^. The ethics committee of the Berlin Chamber of Physicians assessed the ethics of the CORONA-MONITORING local study and provided its approval (Eth-11/20). No patient-specific data was stored or used to perform the method comparison. The SARS-CoV-2-specific antibody immune response was determined by a virus neutralisation (VNT) assay (326 positive, 169 negative, 29 not analysed) and a commercial IgG-specific ELISA (Anti-SARS-CoV-2 ELISA (IgG), Euroimmun, Lübeck, Germany) (355 positive, 148 negative, 21 borderline). Additionally, subpanels of varying size were used to determine the serostatus using multiple commercial assays, namely a sVNT measuring inhibition of receptor binding (GenScript, Piscataway Township, NJ, USA; 307 positive, 205 negative, 12 not determined), as well as multiple commercial serological immunoassays targeting either the SARS-CoV-2 N protein (Abbott Laboratories, Wiesbaden, Germany; 291 positive, 195 negative, 38 not determined; Roche, Penzberg, Germany; 86 positive, 41 negative, 397 not determined) or the S protein (DiaSorin, Dietzenbach, Germany; 326 positive, 160 negative, 38 not determined; Roche, Penzberg, Germany; 87 positive, 40 negative, 397 not determined).

Sera and clinical data from hospitalised patients with COVID-19 were provided by the observational multicentre study “Identification of predictive biomarkers for severe progression of COVID-19” (PreBiSeCov) carried out by the Bundeswehr Hospital Berlin^128^. It was approved by the ethics committee of the Berlin Chamber of Physicians (permit no. Eth-10/20) and prospectively registered at the German Clinical Trials Register (study ID: DRKS00021591). Briefly, immunocompetent adults who required inpatient therapy due to COVID-19 were enrolled at the Bundeswehr Hospitals Berlin, Hamburg, Westerstede, and Koblenz. Serum samples and clinical data were collected at the following time points: on admission to hospital, and 7, 10, 14, and 21 days after onset of symptoms (provided the patients were hospitalised at these dates). The primary endpoint was invasive or non-invasive ventilation within 30 days after admission. A total of 135 patients from the four study centres were enrolled. Since clinical suspicion of COVID-19 was sufficient for inclusion, 14 patients were ultimately found to have other diagnoses than COVID-19 and were subsequently excluded according to the study protocol. Furthermore, 9 patients withdrew consent or were erroneously included, e.g. due to immunosuppression. Thus, 112 patients could be evaluated for the study. Of those, 66% were male and the median age was 60. Leftover sera were provided for the present study (110 individual patients could be analysed for at least one time point during hospitalization. According to WHO criteria^129^, 97 patients had moderate disease (63% male, median age 59), and 13 had severe disease or died (92% male, median age 72).

To test if our bead-based multiplex assay could be used to analyse the immune response after infection and immunisation, an independent panel of 136 sera was used. Of those, sera from 20 volunteers were taken before and after immunisation with either the Pfizer/BioNTech mRNA-based vaccine (Comirnaty^®^) or the vector-based vaccine from AstraZeneca (Vaxcevria^®^) 3 weeks after the first vaccination. Of the sera taken before the first immunisation, one subject of the AstraZeneca group and two subjects of the Pfizer/BioNTech group had been tested positive by PCR for a SARS-CoV-2 infection before the first immunisation. For the Pfizer/BioNTech vaccine, 20 sera were also sampled 3 to 4 weeks after the suggested second vaccination 6 weeks after the first vaccination while, due to the change in vaccination recommendations for the AstraZeneca vaccine, only sera after the first vaccination were available. Additionally, a panel of 20 sera was obtained from patients after infections with the Alpha (B.1.1.7) variant while 16 sera were obtained from patients after infections with the Beta (B.1.351) variant.

Collection, ethical statements and informed consent for the panel of n = 137 sera collected from children aged 1 to 16 years in the scope of the COALA study are described elsewhere^53^. Only age and serological status against SARS-CoV-2 were used as additional information to the multiplex measurements. No other patient data were linked or analysed.

Paired serum samples from 27 patients were collected within the scope of the COViK study described elsewhere^59^. Samples were pseudonymised, information on previous vaccination, time points of symptom onset and blood samplings were used as additional information to determine the time point after symptom onset and vaccination status.

For all serum panels used in this study, written consent by the patients or for minors by a parent and/or legal guardian and/or ethics or data security committees was obtained before the start of the study. Data was only stored and handled pseudonymised without links to patient data apart from the data mentioned in this manuscript. All research was performed in accordance with relevant guidelines/regulations.

### Reagents and antibodies

Protease-free bovine serum albumin (BSA) was obtained from SERVA Electrophoresis (Heidelberg, Germany), 2-(N-morpholino) ethanesulfonic acid (MES) and NaN_3_ from CARL ROTH (Karlsruhe, Germany), NaH_2_PO_4_ and human serum albumin (HSA) from Sigma-Aldrich (Taufkirchen, Germany), Tween^®^ 20 from Merck Millipore (Darmstadt, Germany). MagPlex^®^ Microspheres covering bead regions 7, 8, 15, 20, 26, 33, 36, 42, 48, 54, 55, 59, 62, 67, 81, 82, and 89 were obtained from Luminex^®^ Corporation (Austin, TX, USA), 1-ethyl-3-(3-dimethylaminopropyl)carbodiimide (EDC) and sulfo-*N*-hydroxysuccinimide (NHS) from Thermo Fisher Scientific (Dreieich, Germany). Phosphate buffered saline (PBS, pH 7.3) used in the experiments was produced in-house. Other buffer solutions were prepared according to the xMAP^®^ Cookbook (4th Edition, Luminex^®^ Corporation), namely 100 mM NaH_2_PO_4_ (pH 6.2), 50 mM MES (pH 5.0) and PBS/TBN (PBS containing 0.1% BSA, 0.02% Tween^®^ 20, and 0.05% NaN_3_, pH 7.3) needed for protein coupling to the MagPlex^®^ Microspheres, as well as PBS/B (PBS containing 1% BSA) and PBS/T (PBS containing 0.1% Tween^®^ 20) needed to perform the assay. Streptavidin-R-Phycoerythrin (SA-PE) PJRS27 was purchased from Agilent (Santa Clara, CA, USA).

The following antibodies were used during the study: R-phycoerythrin labelled as well as unlabelled goat anti-human IgG antibody (Fc-γ specific), R-phycoerythrin labelled goat anti-human IgM antibody (µ-specific), and biotinylated goat anti-mouse antibody (H + L specific, MinX Hu,Bo,Ho,Rb,Sw) were obtained from Dianova (Hamburg, Germany), subclass-specific mouse monoclonal antibodies targeting human IgG1 (Cat No: 555868), IgG2 (Cat No: 555873), and IgG4 (Cat No: 555881) were obtained from Becton Dickinson (Heidelberg, Germany) while a mouse monoclonal antibody targeting human IgG3 was obtained from Sigma-Aldrich (clone HP-6050, Cat No: I7260-0.2ML). As certified reference materials, the standard serum EURM-017 was obtained from the Joint Research Centre (European Commission, Geel, Belgium).

### Recombinant antigens

Pertussis toxin (salt free) was obtained from List Labs (Campbell, CA, USA), the S1 domains from the following coronavirus strains expressed in HEK293 cells from Sino Biological Europe were obtained through Hölzel Diagnostika (Cologne, Germany): SARS-CoV-2 (wt Wuhan), MERS-CoV, HCoV-229E, HCoV-NL63, HCoV-HKU1, HCoV-OC43. The S1 domain from SARS-CoV as well as the N protein from SARS-CoV-2 expressed in HEK293 cells by Acro Biosystems were obtained via BIOZOL Diagnostica (Eching, Germany). The RBD from HCoV-229E was obtained from R&D Systems (Minneapolis, MN, USA). Trimeric SARS-CoV-2 spike protein, SARS-CoV-2 RBD and HCoV-OC43 RBD were produced in-house. The SARS-CoV-2 spike protein was constructed as follows: at the C-terminus of the ectodomain (amino acids 1–1211, MFVF- QYIK; GenBank: YP_009724390.1), a T4 fibritin trimerisation motif, an HRV 3C protease cleavage site, a Twin-StrepTag and an 8× HisTag was fused. The S1/S2 furin cleavage site "RRAR" was mutated to "GSAS" and residues K986 and V987 were replaced by proline. The SARS-CoV-2 RBD (amino acids 319–541, RVQP-CVNF; GenBank: YP_009724390.1) was fused at the C-terminus to a 6× HisTag. The HCoV-OC43 RBD (amino acids 335–621, KLNL-QKAN; GenBank: AAR01015) was fused at its C-terminus to a Twin-StrepTag followed by an 8× HisTag. Both RBDs were fused at their N-terminus to the signal peptide of the ACE2 receptor (amino acids 1–17, MSSS-VTAA, Genbank NP_068576). All DNA molecules encoding the recombinant proteins were synthesised as human codon-optimised sequences by GeneArt (ThermoFisher Scientific, Dreieich, Germany) and cloned into the mammalian expression vector pTT5^®^ (pTT^®^ is used under licence from National Research Council of Canada). For protein expression recombinant pTT5^®^ plasmids were used together with polyethylenimine (PEI) to transiently transfect HEK 293-6E cells (National Research Council of Canada) grown as suspension culture in FreeStyle™ F17 expression medium (ThermoFisher Scientific, Dreieich, Germany). SARS-CoV-2 spike protein was purified from the supernatant using a HisTrap™ FF crude column (Cytiva, Freiburg, Germany). SARS-CoV-2 RBD and HCoV-OC43 RBD were both purified with cOmplete™ His-Tag columns (Roche, Penzberg, Germany). HCoV-OC43 RBD was further purified using a Strep-Tactin^®^ resin (IBA, Göttingen, Germany). Purified proteins were stored in PBS and concentration was determined by measuring A_280_ using a NanoPhotometer^®^ (Implen, Munich, Germany). The RBDs from HCoV-strains HKU1 (GenBank: ABC70719.1) and NL63 (GenBank: QE59359.1) were cloned into a pcDNA™3.1 vector (Invitrogen™; ThermoFisher Scientific, Dreieich, Germany) and constructed as follows: AviTag-HKU1-RBD-Twin-His was cloned as SARS-CoV-2 signal peptide (MFVFLVLLPLVSSQ) followed by the AviTag (GLNDIFEAQKIEWHE), GG linker, 311–614 amino acids of HKU1 S protein (GenBank ABC70719.1), followed by GGGS linker, TwinStrep tag (WSHPQFEKGGGSGGGSGGSAWSHPQFEK), and 6× HisTag. AviTag-NL63-RBD-Twin-His was cloned similarly with amino acids 481–616 of NL63 S protein (GenBank QEG59359.1). Cloned constructs were used to transfect FreeStyle™ 293-F cells that were grown in suspension using FreeStyle 293 expression medium (Life Technologies, ThermoFisher Scientific, Dreieich, Germany, #A1435101) at 37 °C in a humidified 8% CO_2_ incubator rotating at 125 rpm. Cells were grown to a density of 2.5 million cells per mL, transfected using PEI (Polysciences Europe GmbH, Hirschberg an der Bergstrasse, Germany, #23966-1, 4 µg/mL in cell suspension) and DNA (1200 ng/mL in cell suspension), and cultivated for 3 days. The supernatants were harvested and proteins purified by His SpinTrap columns according to manufacturer’s instructions (Cytiva, Freiburg, Germany, 95056-290). The eluted protein was transferred to PBS via buffer exchange using Amicon Ultra-4 ultrafiltration column 10 kDa cutoff (Merck Millipore, Darmstadt, Germany, UFC801096). Protein concentration was determined by His-tag specific ELISA using a mouse anti-His-tag antibody (Abcam, Berlin, Germany, #ab18184) and a goat anti-mouse IgG Fc antibody conjugated to alkaline phosphatase (Southern Biotech, Biozol, Eching, Germany, #SBA-1033-04) as detection reagent. Protein production was confirmed by SDS-PAGE and western blot using a mouse anti-His antibody (Abcam, Berlin, Germany #ab18184) and an IRDye 800CW donkey anti-mouse antibody (Li-Cor Biosciences, Bad Homburg vor der Höhe, Germany, #925-32212).

Before implementation into the assay, specific antigens were thoroughly tested for purity and identity by SDS-PAGE followed by Coomassie^®^ brilliant blue staining and MALDI-TOF mass spectrometry (MS) and for some antigens by additional LC–MS/MS with a high-resolution Orbitrap (Q Exactive HF, ThermoFisher Scientific, Bremen, Germany) (see Fig. S1 and Figs. S15–S20).

### Serological multiplex suspension assay

Coupling of MagPlex^®^ microspheres with proteins was done using EDC/NHS-coupling chemistry following the suggestions published in the xMAP^®^ Cookbook (4th Edition, Luminex^®^ Corporation). To this aim, for each coupling reaction 1.5 × 10^6^ microspheres from each bead region were washed in 100 µL *Aqua bidest* and resuspended in 80 µL NaH_2_PO_4_ (pH 6.2) before 10 µL sulfo-NHS (50 mg/mL) and 10 µL EDC (50 mg/mL) were added for 20 min to activate surface carboxyl-groups. Subsequently, beads were washed twice using each 250 µL 50 mM MES (pH 5.0) before 10 µg coronaviral proteins, 20 µg HSA, anti-human-IgG or 7.5 µg pertussis toxin were added in 500 µL MES for 2 h at room temperature. The coupling reaction was blocked for 30 min by incubation with 500 µL PBS/TBN before the microspheres were washed twice using 1 mL PBS/TBN and resuspended in 500 µL PBS/TBN. Using a Neubauer counting chamber, the microspheres were quantified and the concentration adjusted to 1000 microspheres per µL.

To determine IgG antibodies against the coronaviral proteins in the bead-based multiplex assay, 5 µL of serum was diluted in 500 µL PBS/B assay buffer. On each plate, the standard serum EURM-017 was included in two dilutions (1:101 and 1:505), as well as one negative and one positive patient serum measured in a 1:101 dilution. For testing of dried blood spots (DBS), extracts from punches (punch head 4.7 mm) generated on filter paper with dried capillary blood were prepared according to manufacturer’s recommendations (Euroimmun, Lübeck, Germany). Briefly, punches for individual sera were added to a 96-well deepwell plate using a DBS Puncher, Perkin Elmer (PerkinElmer Inc., Waltham MA, USA) and eluted by addition of 250 µL elution buffer (Sample Buffer Blue, Euroimmun, Lübeck, Germany) for 1 h at 37 °C). 210 µL of eluted DBS were transferred to a 96-well plate and measured the same day without any further dilution in the multiplex assay or N- or S-specific SARS-CoV-2 ELISAs (Euroimmun AG, Lübeck, Germany) as described below. The multiplex bead mix containing 1000 beads per region for each antigen tested was freshly prepared for each measurement. 50 µL of the mix was added per well of a polystyrene 96 well flat bottom microplate (Greiner Bio-One, Frickenhausen, Germany) to which 50 µL of diluted serum or standard were added per well and incubated for 1 h at room temperature on a plate shaker (IKA MTS 2/4 digital, IKA-Werke, Staufen im Breisgau, Germany) at 750 rpm. Using a magnetic plate washer (HydroSpeed™, Tecan, Männedorf, Switzerland), plates were washed 3-times with 200 µL PBS/T per well using soak times of 1 min for each washing cycle. Subsequently, 100 µL PE-labelled goat anti human IgG was added per well at a concentration of 1 µg/mL in PBS/B assay buffer and incubated and washed as described before. For IgM detection, 100 µL PE-labelled goat anti human IgM was added instead. Thereafter the washed beads were resuspended in 125 µL sheath fluid (PBS) per well by shaking 1 min at 750 rpm on the plate shaker. To detect the IgG-subclass-specific immune response the assay was performed as described above, but the PE-labelled anti-human IgG antibody was replaced by subclass-specific monoclonal antibodies, which were used at a concentration of 1 µg/mL, followed by detection with biotinylated goat anti mouse IgG antibodies (1 µg/mL, 45 min at room temperature) and PE-labelled streptavidin (1 µg/mL, 30 min at room temperature) with intermittent washing steps as described above. Finally, signals were analysed on a Bio-Plex 200 instrument (Bio-Rad, Munich, Germany) using normal detector gain (RP1 target) collecting data from at least 50 beads per region. Raw data was exported into a Microsoft Excel^®^ sheet and further processed and analysed as described below.

### Reference assays

To calculate cut-off values for the SARS-CoV-2-specific antigens and compare the assay performance of the multiplex assay with different reference methods, several commercial assays as well as a virus neutralisation (VNT) assay were performed in parallel. To this aim the neutralising titre of 495 sera from the hotspot panel was tested in a plaque reduction neutralisation assay as described previously^130^. A wild-type SARS-CoV-2 strain was used for the VNT, which was isolated during the first outbreak that occurred in Germany^43^. Sera with a 50% plaque reduction neutralisation titre above or at a 1:20 dilution were considered positive. A total of 503 sera that were tested in the commercial SARS-CoV-2 S1-specific ELISA (Euroimmun, Lübeck, Germany) as described before^41^ were included for the method comparison (cut-off value ELISA ratio > 1.1 positive, ELISA ratio < 0.8 negative, borderline results excluded). Additionally, 512 sera from the hotspot panel were tested in a SARS-CoV-2 surrogate virus neutralisation test (sVNT) (GenScript; cut-off value ≥ 30% inhibition positive), 486 sera were tested in a SARS-CoV-2 S-specific anti-SARS-CoV-2-IgG CLIA for Liaison^®^ (DiaSorin, Saluggia, Italy; cut-off ratio ≥ 15 positive) or anti-SARS-CoV-2-IgG CMIA for ARCHITECT (Abbott Ireland, Sligo, Ireland; cut-off ratio ≥ 1.4 positive), 127 sera were also tested in two automated electro-chemiluminescence immunoassay (ECLIA) targeting the S or the N protein from SARS-CoV-2 (Roche, Penzberg, Germany; cut-off ratio > 1 positive). For the validation of the IgM detection, an N protein specific ELISA was obtained from Euroimmun while an RBD-specific IgM-ELISA was obtained from Merck Millipore (Darmstadt, Germany, # EZRBDM-120K). All assays were performed and analysed as published or as recommended by the suppliers. For simplicity, all non-multiplexed binding assays are referred to as serological immunoassays throughout the manuscript.

### Data analysis

All data analysis was done using the statistical programming language R (version 4.1.2)^131^. For data handling and wrangling, the packages rio (version 0.5.27), tidyverse^132^, and reshape^133^ were used, for plotting the packages corrplot (version 0.92), ggthemes (version 4.2.4), and scales (version 1.2.1) were used. Calculation of the population-based cut-off value based on the distribution of the binding signals was performed using the package cutoff (version 1.3), while the multiple method comparison to determine cut-off values using method comparisons with different reference methods was done using the package pROC^51^.

To minimise batch-to-batch variability, all SARS-CoV-2-specific binding signals were normalised to the corresponding binding signals of the standard serum EURM-017 measured at a 1:505 dilution on each plate. To this aim, the log10 of the ratio between the binding signal on a SARS-CoV-2 specific antigen for each test serum and the binding signal of EURM-017 measured on the same plate for the same antigen was calculated and transformed by adding 3. By that, the initial distribution of the binding signals was retained while all normalised signals remained positive, which was necessary for fitting normal distributions to determine the population-based cut-off values.

A population-based cut-off was calculated by using finite mixture models to separate the two peaks in the bimodal data distribution of the SARS-CoV-2-specific signals. The method has been described before^47^ and was implemented in the cutoff package, which can be installed via GitHub (https://github.com/choisy/cutoff). Briefly, using the Expectation–Maximisation algorithm^134^ a finite mixture model assuming two normal distributions was fit over the normalised binding signals for each SARS-CoV-2-specific antigen for the n = 524 sera from the hotspot studies containing positive and negative sera as well as the n = 100 pre-pandemic sera containing only negative sera. A cut-off value was calculated by computing the probability for each data point to belong to either one of the two distributions from the fitted finite mixture model assuming a type-I-error of 0.05. This approach was possible as two clearly separated populations consisting of low signals in the naïve population and high signals in recently infected individuals were detected in the data. Similarly, population-based cut-off values were determined for each of the four HCoV-strains based on the signal distribution in the 100 pre-pandemic sera. HCoV-specific signals were normalised analogous to the SARS-CoV-2 specific signals with the only exception that the 1:101 diluted standard serum EURM-017 measured on each plate was used as reference point.

Cut-off values based on method comparisons were calculated using the pROC package. To this aim, normalised binding signals for the SARS-CoV-2-specific antigens of the multiplex assay were used as predictor to calculate receiver operator characteristics using classifications in positive and negative by one of the following assays as response: VNT, sVNT based on inhibition of receptor-binding (GenScript), commercial serological immunoassays targeting either S (Euroimmun, DiaSorin, Roche) or N protein (Roche, Abbott). Additionally, generalised linear models were modelled for each reference method considering all four SARS-CoV-2-specific antigens using the following formula: reference predictor ~ normalised S1 signals + normalised trimeric spike signals + normalised RBD signals + normalised N signals, family = “binomial”. Confidence intervals were calculated using 2000 stratified bootstrap replicates of the generated ROC curves. As described above, the following normalized cut-off values were determined for the SARS-CoV-2-specific antigens in our bead-based multiplex assay to discern positive from negative sera: for TriS 2.76 (95% confidence interval (CI) ranging from 2.70 to 2.90), for S1 2.48 (2.42–2.53), for RBD 2.51 (2.46–2.57), and for N 2.59 (2.52–2.72), respectively.

To determine a cut-off value to discern early from late time points based on IgG/IgM-ratios, those ratios were calculated from normalised IgG and IgM data for each of the four SARS-CoV-2-specific antigens. Normalisation was performed as described above for the SARS-CoV-2-specific antigens. A probit analysis was performed by generating a generalised linear model using the following formula: glm(time point ~ IgG/IgM-ratio, family = binomial(link = "probit")) with the early time point after symptom onset set to 0 and the late time point after symptom onset set to 1.

### Supplementary Information

Supplementary Information.

## Data Availability

All data generated or analysed during this study are included in this published article and its Supplementary Information files.
