# Supplementary material for: A bead-based multiplex assay covering all coronaviruses pathogenic for humans for sensitive and specific surveillance of SARS-CoV-2 humoral immunity
Source: Sci Rep. 2023 Dec 9;13:21846. doi: 10.1038/s41598-023-48581-9 (PMC10710470; doi:10.1038/s41598-023-48581-9)
Supplement: Supplementary file 1 — Supplementary Information. [file 41598_2023_48581_MOESM1_ESM.docx]

Supplementary Information

A bead-based multiplex assay covering all Coronaviruses pathogenic for humans for sensitive and specific surveillance of SARS-CoV-2 humoral immunity

**Daniel Stern^1#,*^, Tanja C. Meyer^1#^, Fridolin Treindl^1^, Hans Werner Mages^1^, Maren Krüger^1^, Martin Skiba^1^, Jan Philipp Krüger^2^, Christian M. Zobel^3^, Maximilian Schreiner^3^, Marica Grossegesse^4^, Thomas Rinner^4^, Caroline Peine^5^, Anna Stoliaroff-Pépin^5^, Thomas Harder^5^, Natalie Hofmann^4^, Janine Michel^4^, Andreas Nitsche^4^, Silke Stahlberg^6^, Antje Kneuer^6^, Anna Sandoni^6^, Ulrike Kubisch^6^, Martin Schlaud^6^, Annette Mankertz^7^, Tatjana Schwarz^8^, Victor M. Corman^8,9,10^, Marcel A. Müller^8^, Christian Drosten^8^, Kathrin de la Rosa^11,12^, Lars Schaade^13^, Martin B. Dorner^1^, and Brigitte G. Dorner^1,^***

# Supporting information

## Algorithm for determination of the serostatus

For each of the four antigens (S1, RBD, TriS, N), the serostatus (positive or negative) was determined by using the robust cut-off values as the mean of individual cut-off values from suitable reference assays as described in the manuscript. An area of uncertainty was calculated as the mean of the upper and lower limits of the 95% confidence intervals as determined from the ROC analysis for the corresponding reference assays. Values within the area of uncertainty were labelled “borderline” in addition to being either positive or negative when above or below the cut-off. For a combined serostatus for all S-specific antigens, different possible combinations of either “borderline” or clear positive and negative results had to be considered to come up with only four final classifications: positive, negative, borderline positive, and borderline negative. Samples with congruent results for all three S-specific results were classified identically as the individual results. Samples with congruent outcomes (all positive or all negative) were classified as clear positive or negative as long as only one S-specific antigen was borderline, else they were classified as borderline on S. If two S-specific antigens of three were positive or negative, samples with one borderline result were classified as borderline positive or borderline negative, else as being clear positive or negative. Samples with only one positive S-specific antigen and two negative antigens were classified as borderline negative, when the one positive sample was clearly positive, else as negative.

# Supporting figures


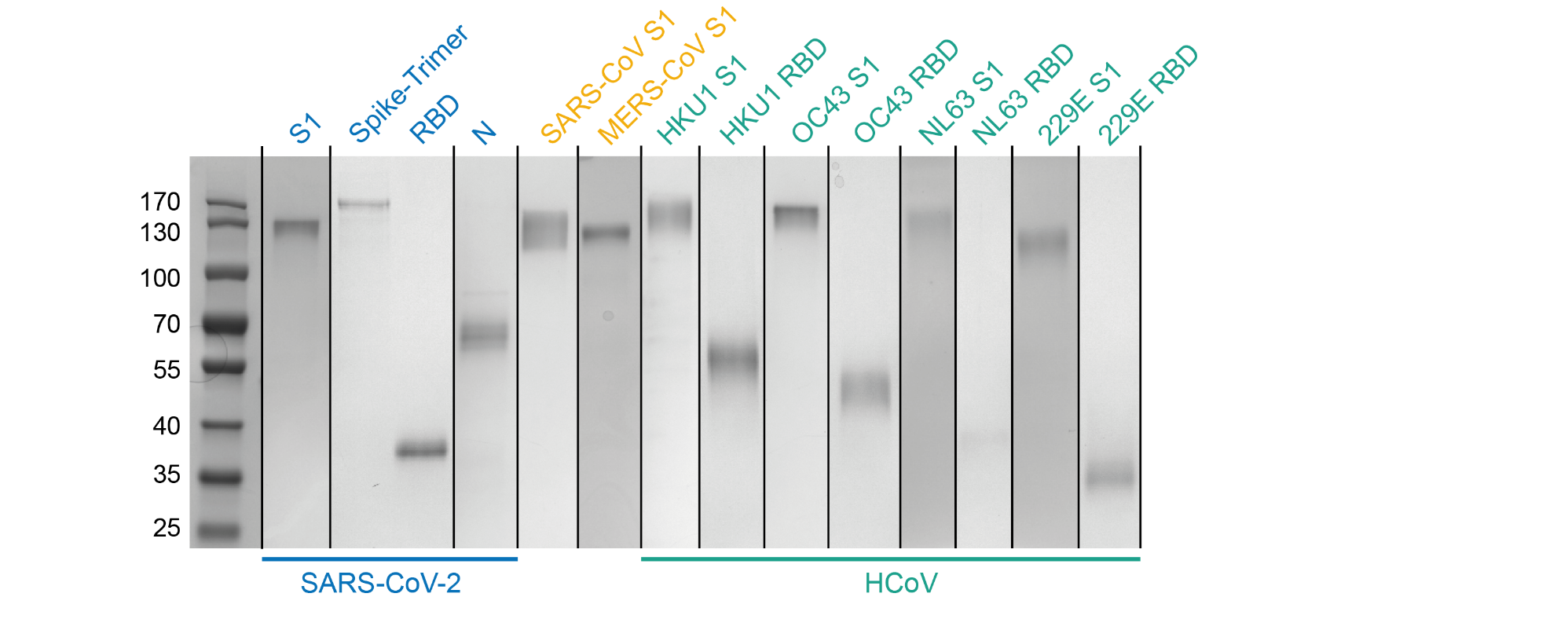


**Figure S1.** Coomassie staining of commercial and in-house produced antigens implemented into the multiplex-assay (Table S1). For each antigen, either 750 ng per lane (RBDs) or 1000 ng per lane (all other) were separated on 10% precast polyacrylamide gels (10% Mini-PROTEAN® TGX™ Precast Protein Gels, BioRad, Munich, Germany) and stained using a colloidal Coomassie staining protocol as described [1-3]. Lanes from different gels are combined or rearranged for better comparison (vertical black lines separate different gels). Uncropped images for each gel are shown in figures S15 to S20.


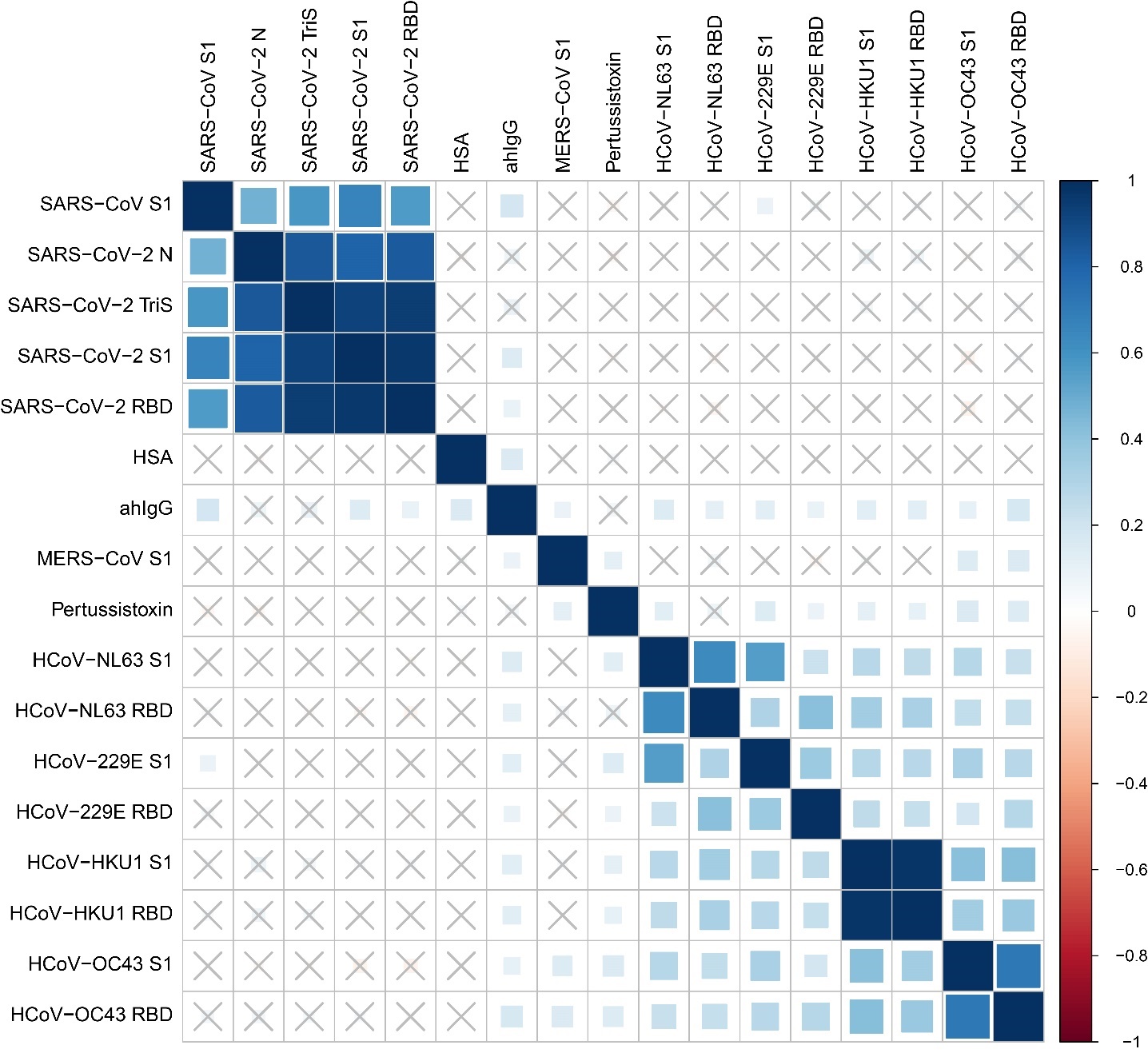


**Figure S2.** Plot of Pearson correlation coefficients between mean fluorescent intensities for the different antigens in a panel of 524 sera sampled during epidemiological investigations of COVID-19 outbreaks in Germany as measured in the 17-plex bead-based serological suspension-assay. × indicates a non-significant statistical correlation, blue represents a positive correlation, red a negative correlation as indicated on the scale on the right side.


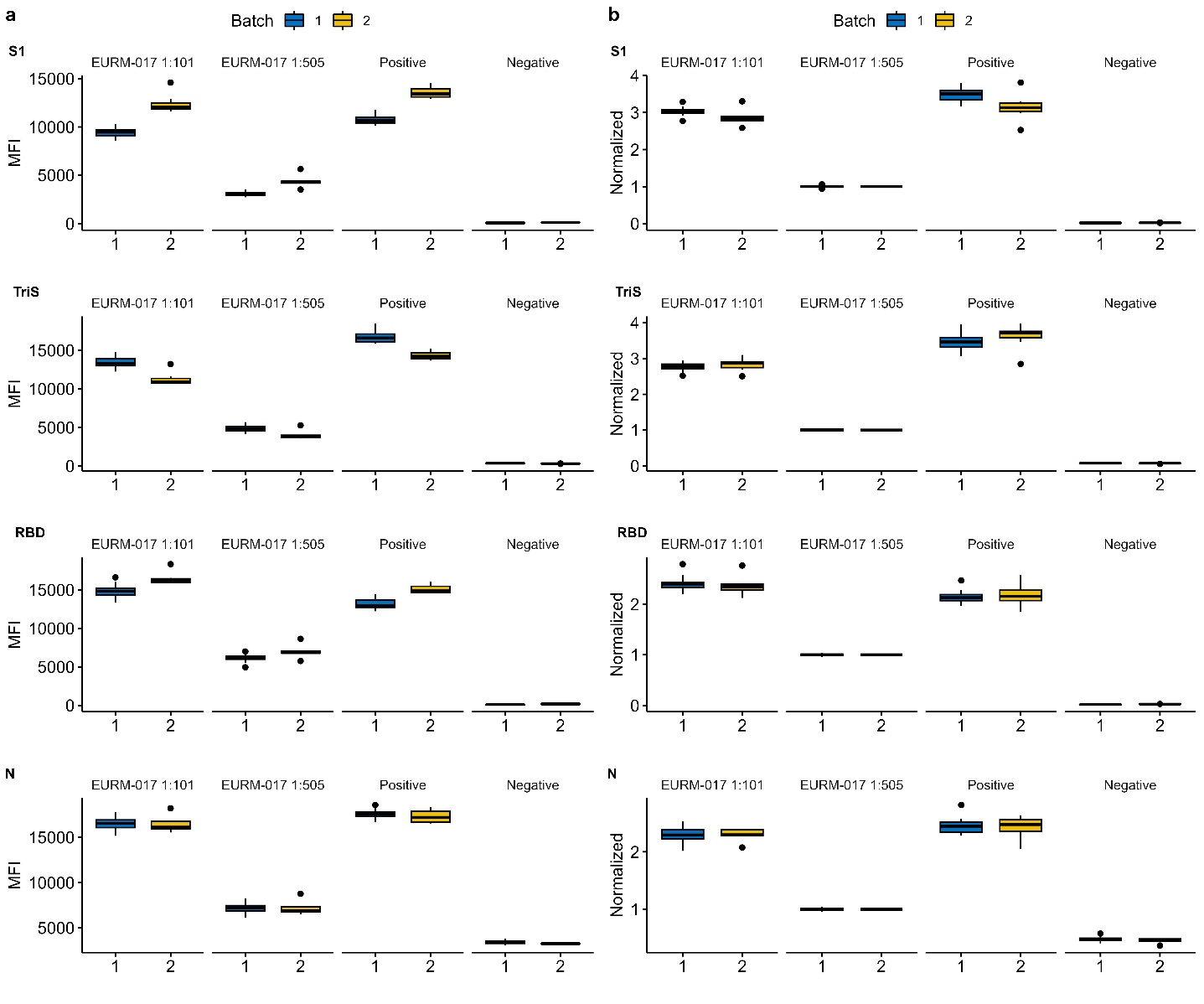


**Figure S3. Impact of normalization on batch-to-batch variability.** a. Binding signals (MFI) of IgG antibodies to two different batches of xMap-beads (Batch 1, n = 13 plates; batch 2, n = 7 plates) coupled with the S1, trimeric spike (TriS), RBD, and N protein from SARS-CoV-2 were determined by measuring two dilutions (1:101 and 1:505) of the international reference serum EURM-017 and a 1:101 dilution of one positive and one negative serum. b. Binding signals for the same sera and antigens as shown in subpanel a are normalized to the binding signals of the international reference serum EURM-017 measured at a 1:505 dilution on each of the 20 plates measured (Batch 1, n = 13; batch 2, n = 7). Normalized binding signals were log10-transformed and shifted to positive values by adding an artificial value of 3.


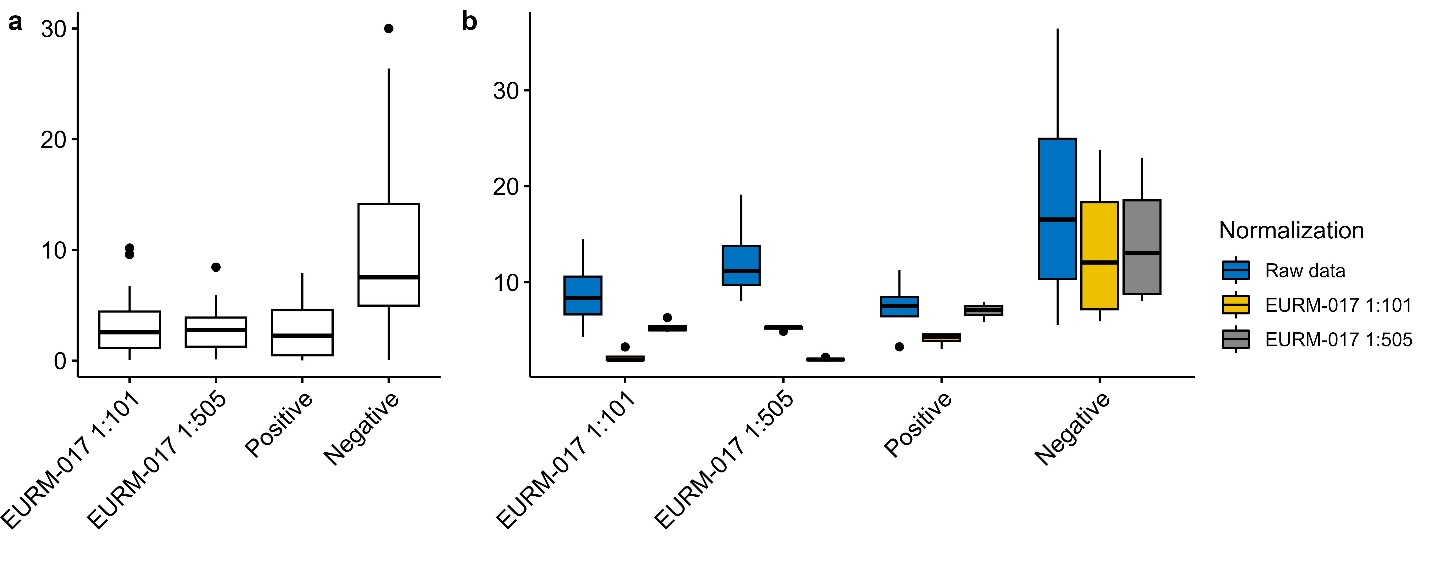


**Figure S4. Intra- and interassay variability in a small panel of four sera over 20 independent measurements in duplicates.** a. Intraassay variability in percent between two duplicate measurements in 20 independent experiments for the reference serum EURM-017 measured in two dilutions (1:101 and 1:505), a positive and a negative serum without normalization applied to the data. b. Interassay variability of the before mentioned sera without normalization (raw data) or normalized to signals of the reference serum EURM-017 measured in two dilutions). Normalization significantly reduced the interassay variability below 10% or 20% for the negative serum.

**
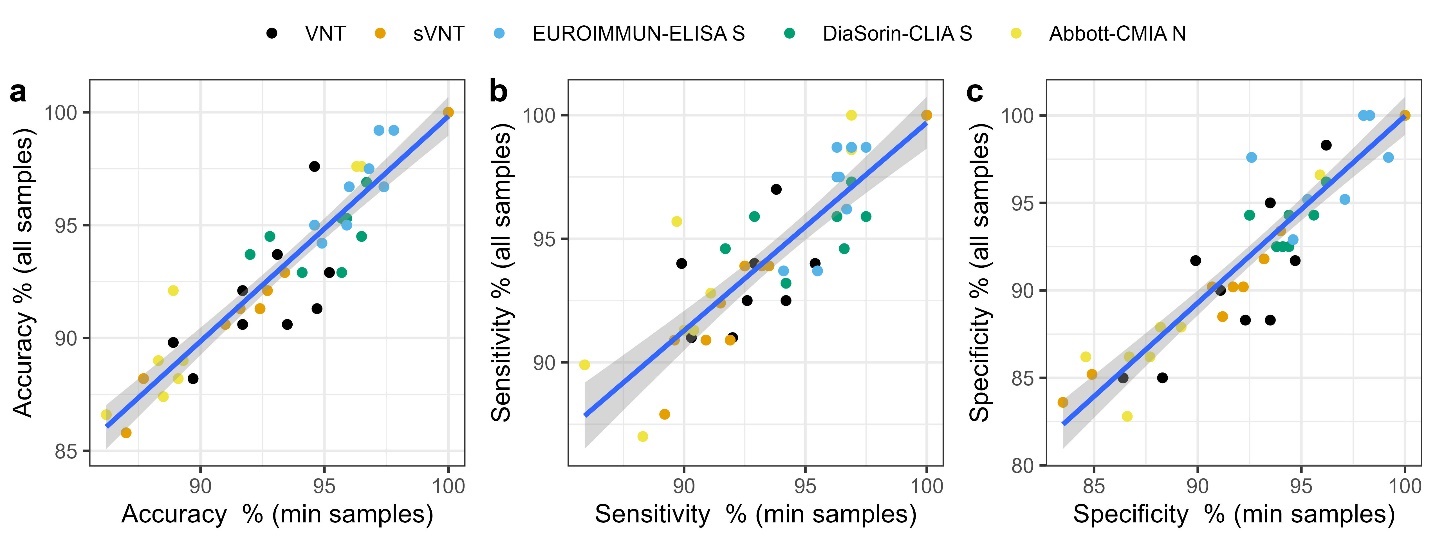
**

**Figure S5. Linear regression between results of ROC analysis performed on the common minimum panel of samples (n = 127) tested in the Roche assays or all samples as shown in Table S2.** Data is shown for accuracies (a), sensitivities (b) and specificities (c) as determined for the comparison between the individual assays (bead-based multiplex assay with SARS-CoV-2-specific antigens, VNT, sVNT, EUROIMMUN-, DiaSorin-, and Abbott-CMIAs) and the color-coded reference assays. Roche-N and Roche-S ELISA were excluded as those assays defined the minimum panel of samples and hence perfect agreement was expected. Pearson correlation coefficients were calculated to 0.94 for accuracy, to 0.89 for sensitivity, and to 0.92 for specificity.

**
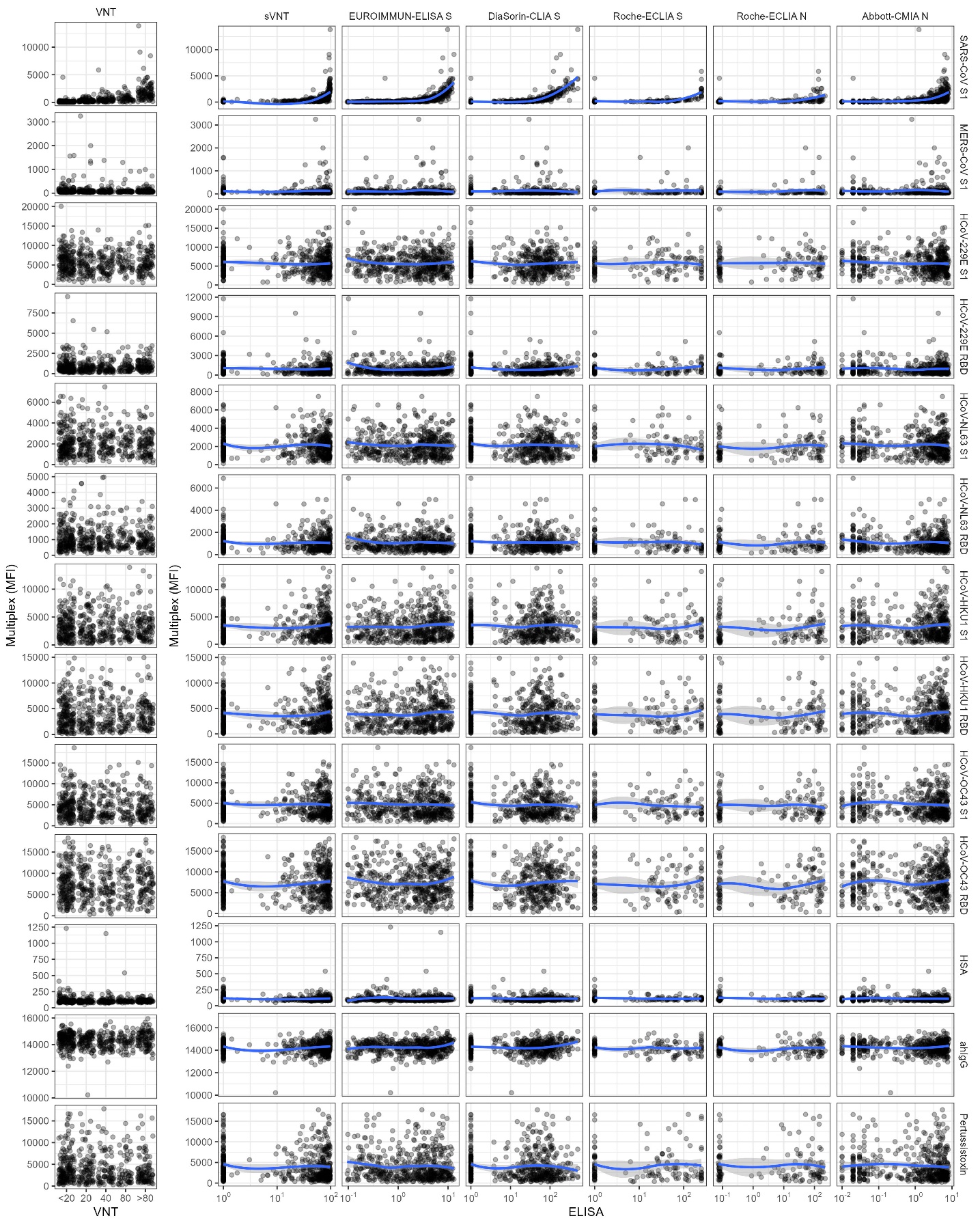
**

**Figure S6. Scatterplots for comparison between SARS-CoV-2 specific reference assays and non-SARS-CoV-2 antigens (targeting SARS-CoV, MERS and low pathogenic coronavirus strains and controls) implemented in the multiplex-assay.** All assays (VNT, sVNT, commercial serological immonassays) were specific for SARS-CoV-2 or tested with SARS-CoV-2 (VNT). Blue regression lines have been fitted using local polynomial regression fitting (locally estimated scatterplot smoothing, loess).

**
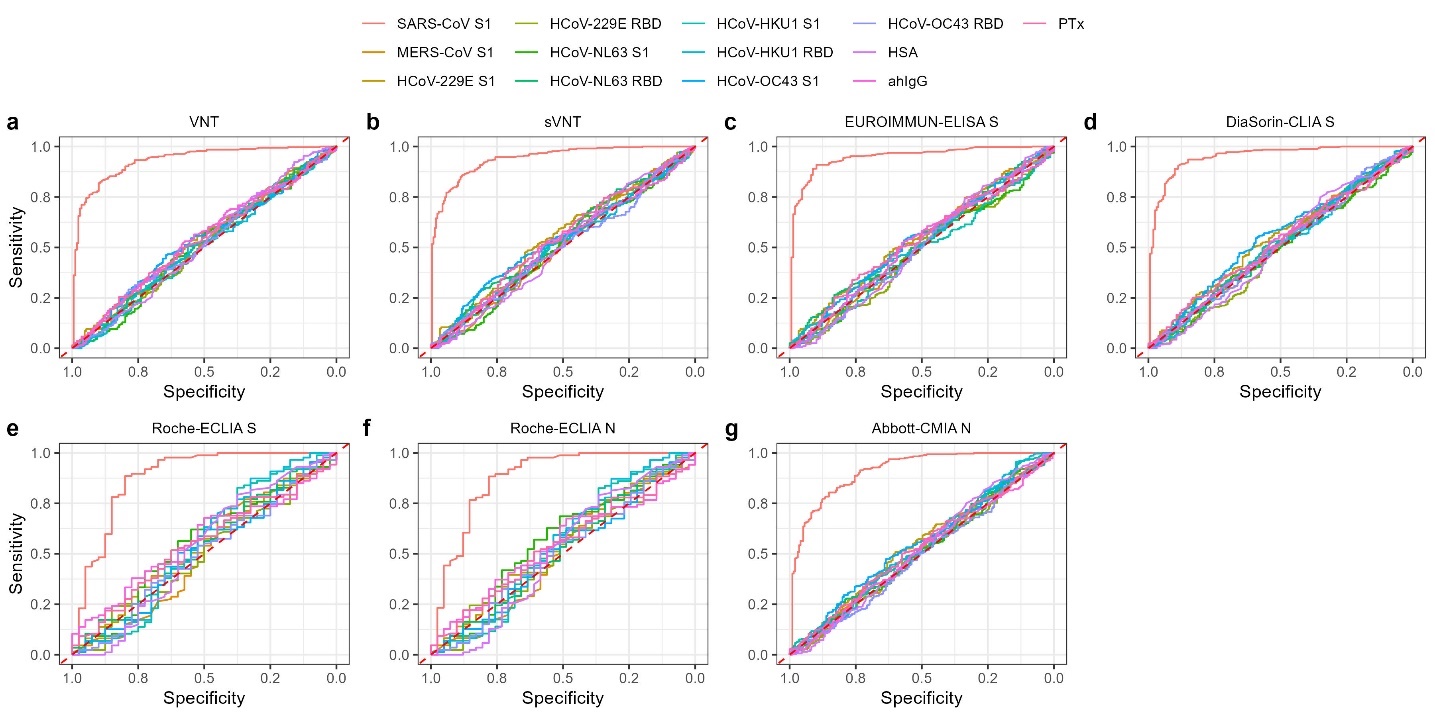
**

**Figure S7. ROC analysis for non-SARS-CoV-2 antigens in the bead-based multiplex assay.** Shown are ROC plots for the determination of cut-off values by comparison with the VNT assay (a), sVNT (b), EUROIMMUN-ELISA S (c), DiaSorin-CLIA S (d), Roche-S- (e), Roche-N- (f) and Abbott-N (g) ELISAs.

**
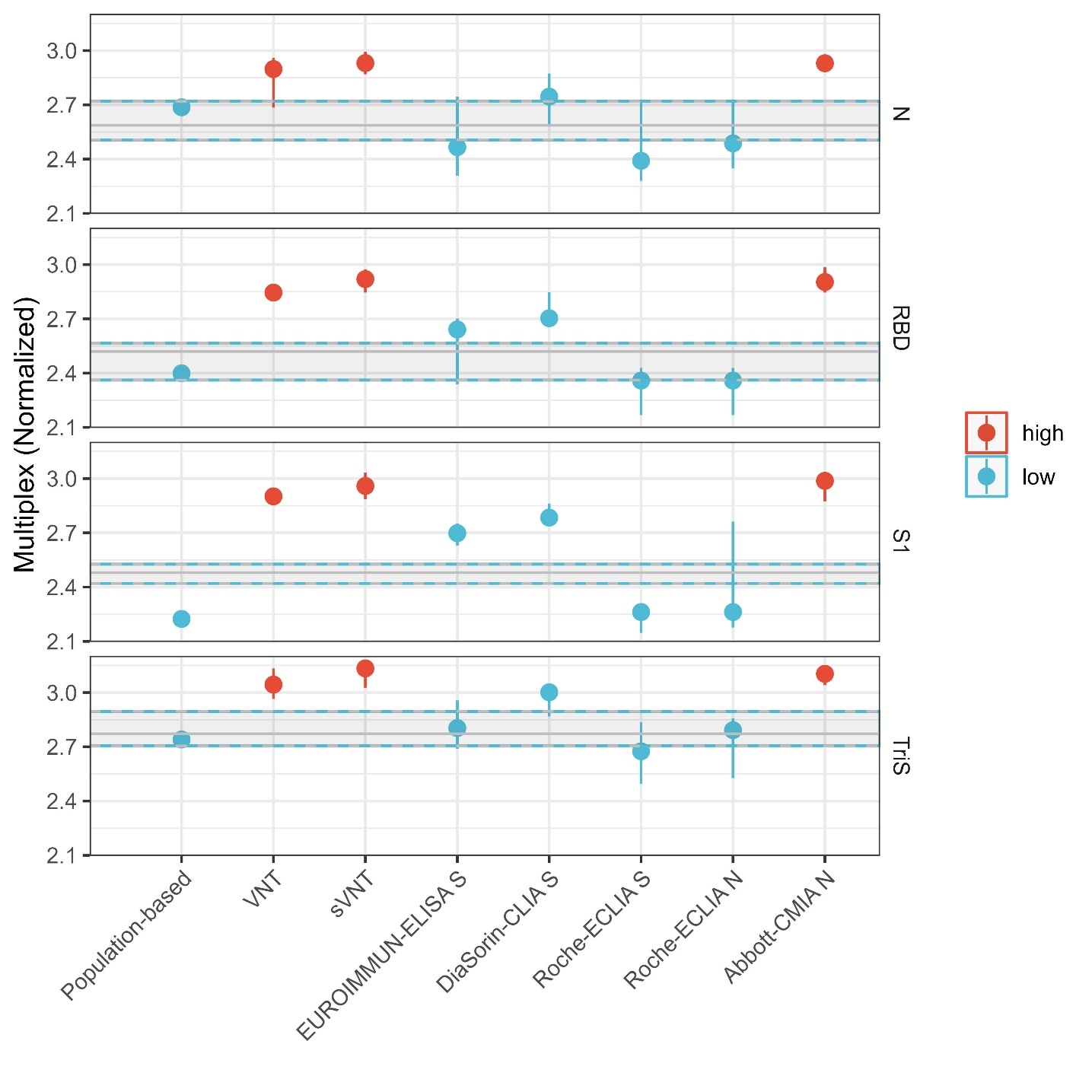
**

**Figure S8 Comparison of normalized cut-off values based on the different reference methods for each of the four SARS-CoV-2-specific antigens implemented in the bead-based multiplex assay.** Shown are cut-off values and 95% confidence intervals as determined by the population-based approach or ROC-analysis for neutralization or surrogate-neutralization assays (VNT, sVNT) or ELISA-based approaches (EUROIMMUN-S, DiaSorin-S, Roche-S, Roche-N, Abbott-N). Grey horizontal lines and shaded areas limited by dashed blue lines indicate median cut-off values calculated for each antigen from the population-based cutoff and the S-specific ELISAs (EUROIMMUN, DiaSorin, Roche) for the S1, TriS, or RBD antigens or from the population-based cut-off and the N-specific ELISA by Roche for the N antigen. High cut-off values (red) were excluded from calculations. Upper and lower limits were calculated as the median values.


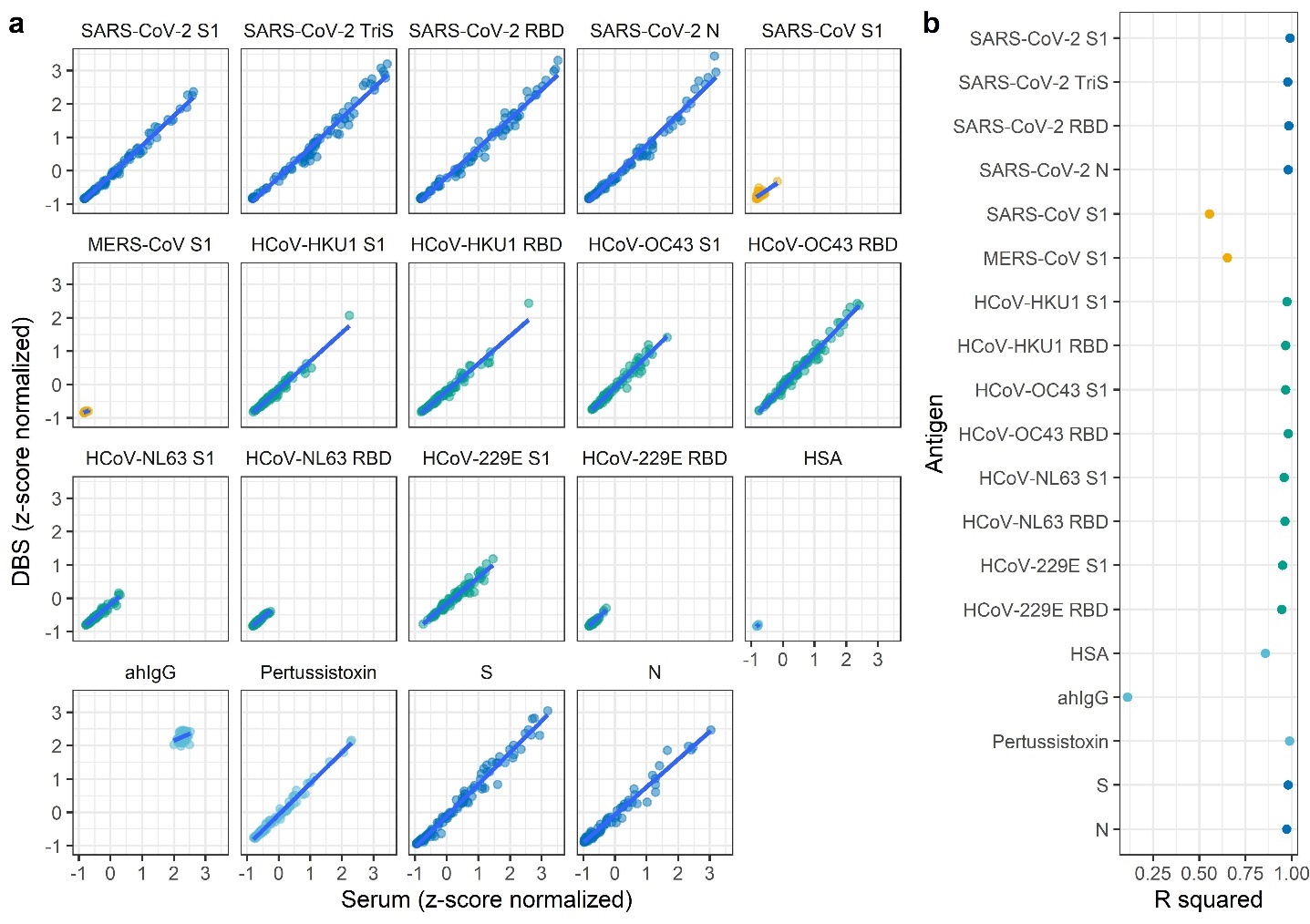


**Figure S9.** a. Multiple comparison between detection of IgG antibodies from serum or dried blood spots (DBS) for all antigens implemented in the bead-based multiplex assay or by N- or S-specific commercial ELISAs (EUROIMMUN, S and N). Linear regressions (blue lines) were calculated for all antigens and assays between z-score normalized signals. b. R^2^ values are shown for better comparability.

**
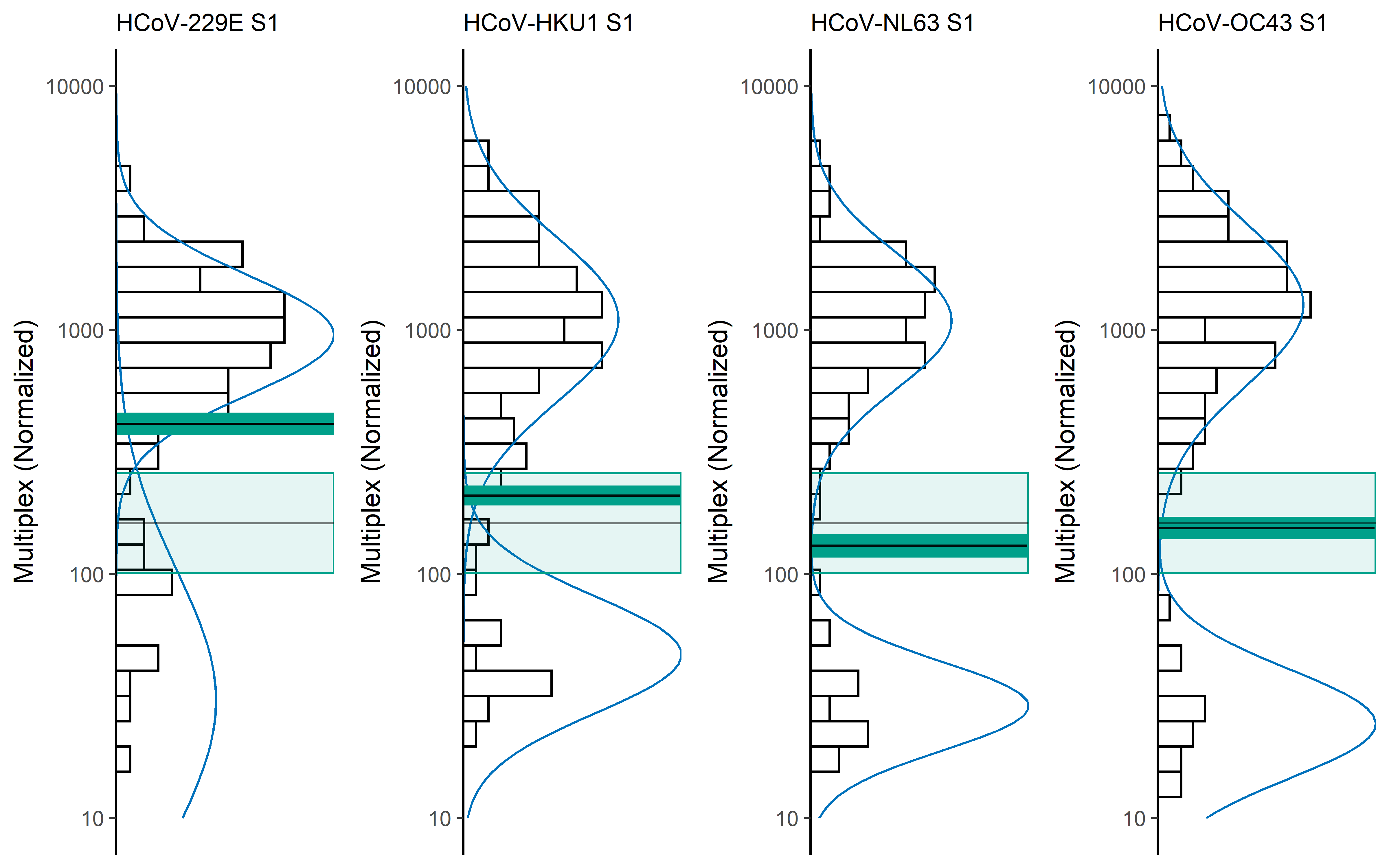
**

**Figure S10.** Determination of a population-based cut-off for the four endemic HCoV-strains. Two normal distributions were fit over the signal distribution for the S1 antigens of HCoV-strains 229E, HKU1, NL63, and OC43 in the panel of n = 100 pre-pandemic sera. Individual cut-off values (narrow dark-green band with central black line) were calculated for each HCoV strain. For a robust cut-off value for all four strains, the mean ± 1.96 × standard deviation of the individual cut-off values for strains HKU1, NL63, and OC43 was calculated (light grey line and light green area). The cut-off value for strain 229E was excluded from the calculation as it was fitted too high due to a broad standard distribution over the low-signal values.


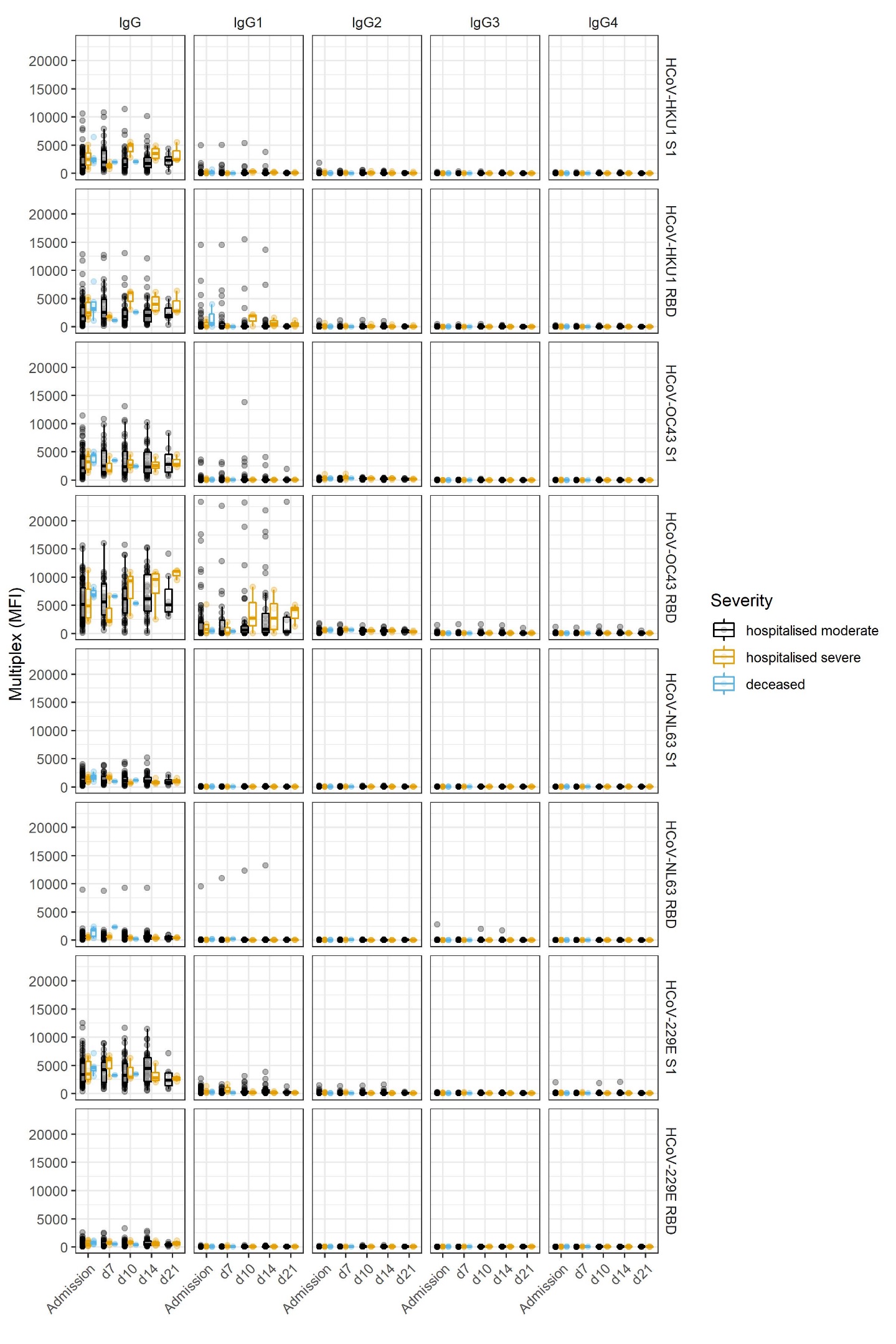


**Figure S11.** Time course of IgG response in a panel of patients hospitalized with moderate or severe symptoms as determined by HCoV-specific antigens in the bead-based multiplex assay. No clear trend is seen with increasing time after admission. Only a slight increase of signals on the HCoV-OC43 RBD, which is, however, not mirrored by an increase on the OC43 S1-specific signals.


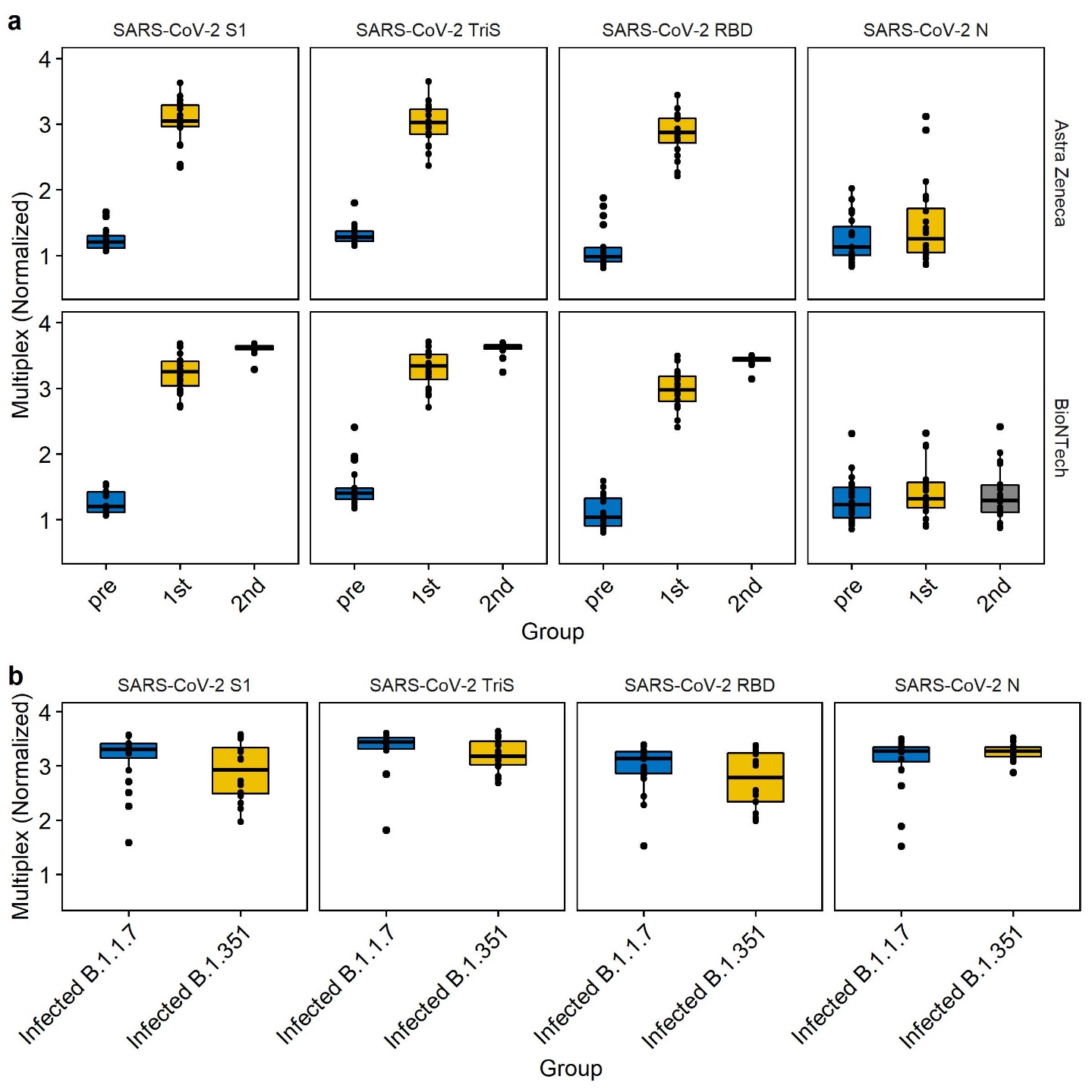


**Figure S12.** Immune response in a panel of immunised or infected individuals as determined by the bead-based multiplex assay. a. Binding to the four SARS-CoV-2-specific antigens before (pre), three weeks after the first (1st) or three to four weeks after the second (2nd) immunisation with either Astra Zeneca (Vaxzevria) or BioNTech (Comirnaty) vaccines. No data was available for the second immunisation with the Astra Zeneca vaccine. Sera from patients who were already infected before immunisation were excluded from the first timepoint pre-immunisation. b. Immune response after PCR-confirmed infection with SARS-CoV-2 variants Alpha (B.1.1.7) or Beta (B.1.351). Sera were taken at least 8 days after the positive PCR-test.


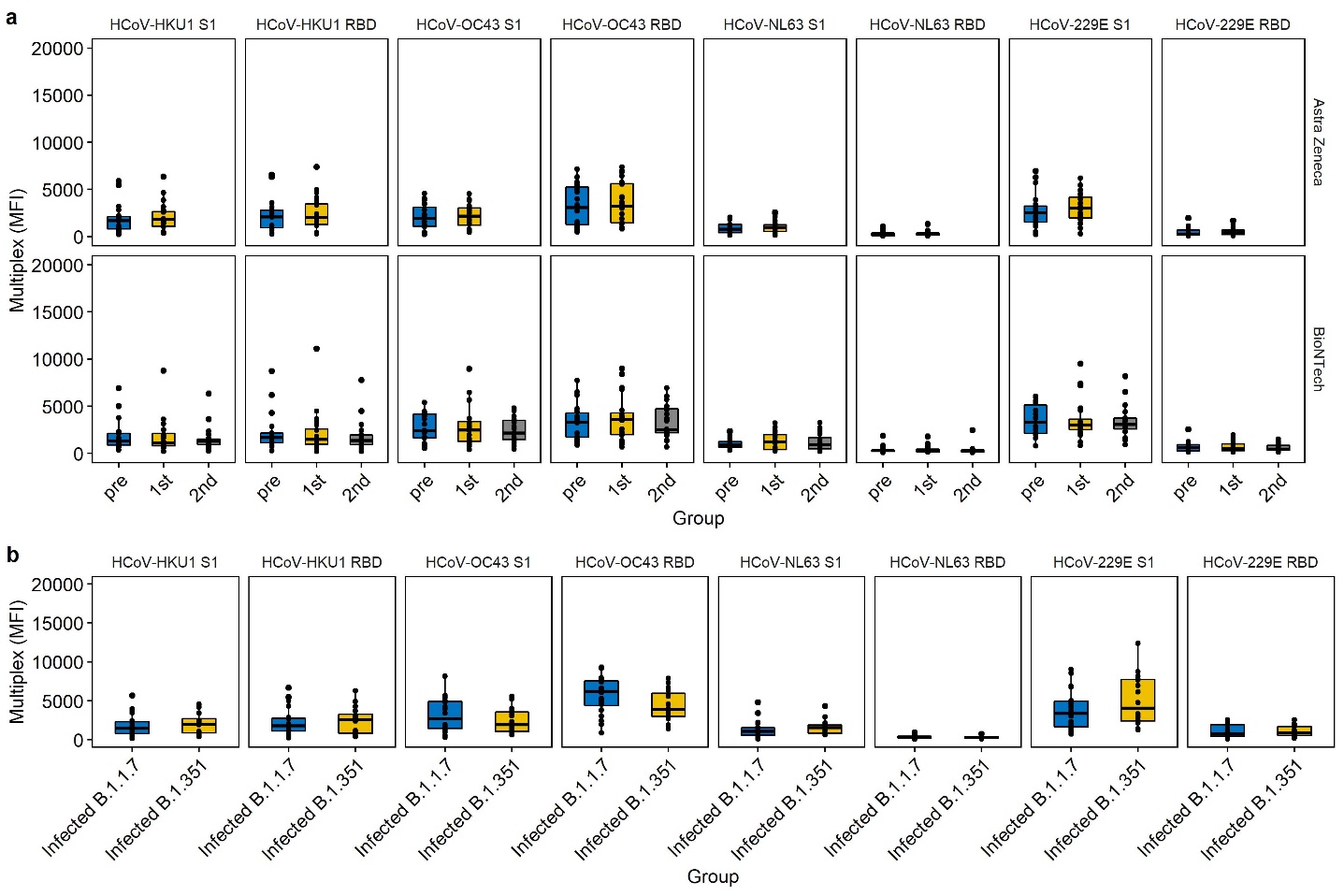


**Figure S13.** Binding signals of sera of immunised or infected patients to HCoV-specific antigens in the multiplex-assay. a. Binding to the S1 or RBD of HCoV strains HKU1, OC43, NL63, or 229E after immunisation with either the Astra Zeneca vaccine (Vaxcevria^®^) or the BioNTech vaccine (Comirnaty^®^) before (pre), three weeks after the first (1^st^) or three to four weeks after the second (2^nd^) immunisation. b. Binding to HCoV-specific antigen in the bead-based multiplex assay after infection with the Alpha (B.1.1.7) or Beta (B.1.351) variant.


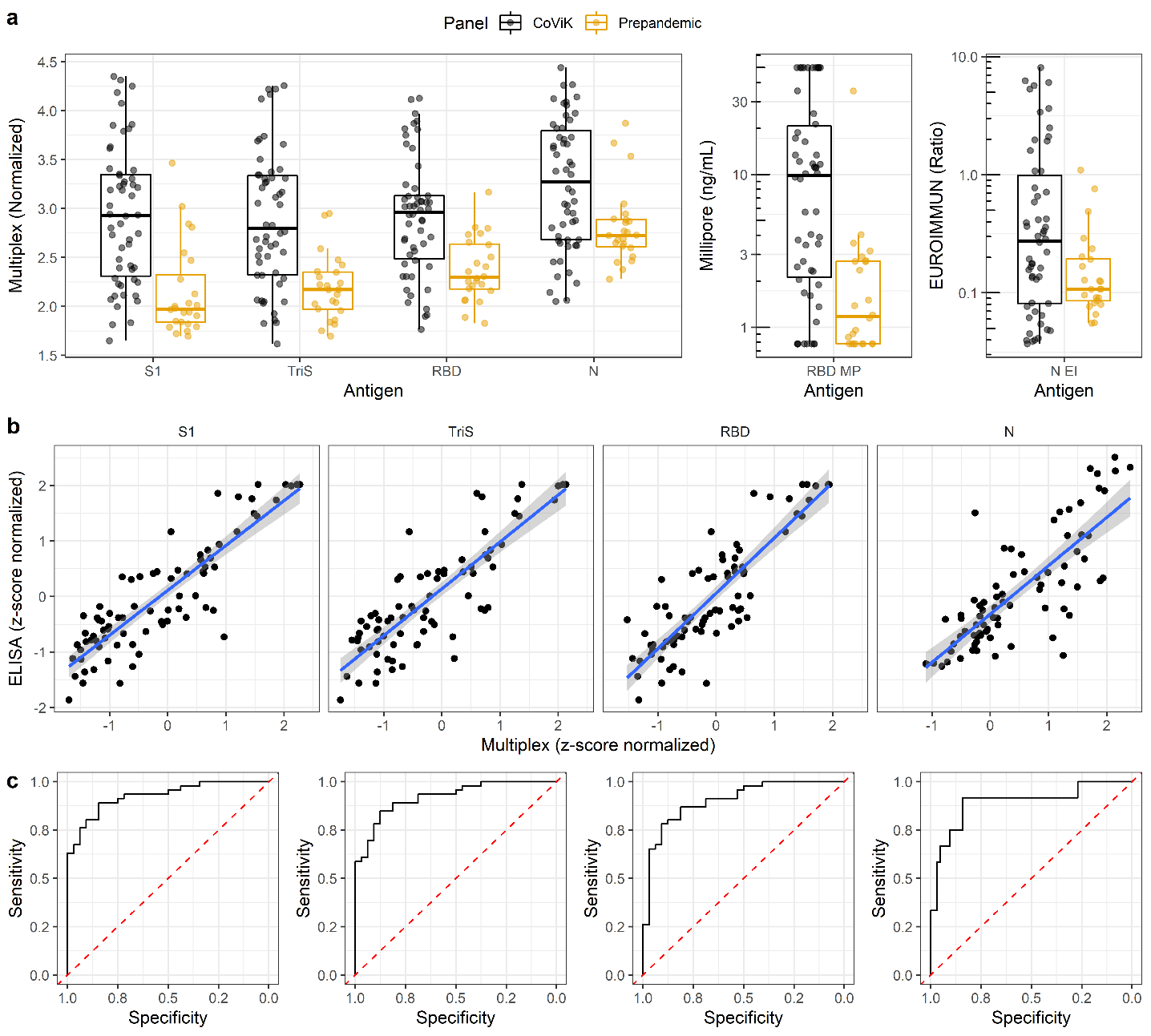


**Figure S14.** Validation and determination of cut-off values for the detection of IgM antibodies against SARS-CoV-2 specific antigens by method comparison with two commercial ELISAs. a. Signal distribution for the IgM-detection in a panel of 78 sera (27 paired sera from CoViK study, each serum sampled early and late after infection, leading to 54 sera, and 24 prepandemic sera) for each of the four SARS-CoV-2-specific antigens implemented in the bead-based multiplex assay. b. Corresponding signal distribution on two commercial IgM-specific ELISAs (RBD-specific, Merck Millipore, N-specific EUROIMMUN EI). b. Linear regression between z-score normalized signals of the multiplex assay and the IgM-specific commercial ELISA. For the S-specific antigens in the multiplex assay (S1, TriS, RBD, N), the RBD-specific ELISA was used for comparison, for the N antigen in the multiplex assay, the N-specific ELISA was used for comparison. c. ROC analysis for the method comparison between the corresponding multiplex antigens in figure b to determine the assay performance parameters and cut-off values for the SARS-CoV-2-specificic antigens for IgM binding.

**Figure S15**. Full-length gel for different tested antigens. Lanes 1 and 5 were included in figure S1 as marker and as lane 2, respectively.

**Figure S16.** Full-length gel for different tested antigens. Lanes 2 and 3 were included in figure S1 as lanes 12 and lane 14, respectively.

**Figure S17.** Full-length gel for different tested antigens. Lanes 3 was included in figure S1 as lane 7.

**Figure S18.** Full-length gel for different tested antigens. Lanes 2 was included in figure S1 as lane 8.

**Figure S19.** Full-length gel for different tested antigens. Lanes 2, 3, and 4 were included in figure S1 as lanes 5, 10, and 6.

**Figure S20.** Full-length gel for different tested antigens. Lanes 2, 3, 4, 5, 6, and 7 were included in figure S1 as lanes 3, 4, 15, 13, 11 and 9.

**Table S1.** Antigens used in this study to establish a 17-plex serological suspension-assay for detection of the humoral immune response against SARS-CoV-2 and related Coronaviruses pathogenic to humans.

| Viral strain | Target protein | Supplier/Source | Order number | Sequence | Host | Reference strain | Calculated MW (kDa) |
| --- | --- | --- | --- | --- | --- | --- | --- |
| SARS-CoV-2 | S1 domain | Sino Biological | 40591-V08H | Val16-Arg685 | HEK293 | YP_009724390.1 | 76.5 |
| SARS-CoV-2 | Trimeric Spike | Recombinant expression in-house | – | Met1-Lys1211 | HEK293 | YP_009724390.1 | 424.3 (Trimer) |
| SARS-CoV-2 | RBD domain | Recombinant expression in-house | – | Arg319-Phe541 | HEK293 | YP_009724390.1 | 25.9 |
| SARS-CoV-2 | Nucleoprotein (N) | Acro Biosystems | NUN-C5227 | Met1 - Ala419 | HEK293 | QHO62115.1 | 47.3 |
| SARS-CoV | S1 domain | Acro Biosystems | S1N-S52H5 | Ser14 - Arg667 | HEK293 | AAP13567.1 | 74.9 |
| MERS-CoV | S1 domain | Sino Biological | 40069-V08H | Tyr18-Glu725 | HEK293 | AFS88936.1 | 79.9 |
| HCoV-HKU1 | S1 domain | Sino Biological | 40602-V08H | Ala13-Arg756 | HEK293 | Q0ZME7.1 | 85.2 |
| HCoV-HKU1 | RBD domain | Recombinant expression in-house | – | Val311-Ile614 | HEK293 | ABC70719.1 | 41.3 |
| HCoV-OC43 | S1 domain | Sino Biological | 40607-V08H1 | Phe13-Leu794 | HEK293 | AVR40344.1 | 89.0 |
| HCoV-OC43 | RBD domain | Recombinant expression in-house | – | Lys335-Asn621 | HEK293 | AAR01015 | 35.6 |
| HCoV-229E | S1 domain | Sino Biological | 40601-V08H | Cys16-Asn536 | HEK293 | APT69883.1 | 58.3 |
| HCoV-229E | RBD domain | R&D Systems | 10612-CV | Ser292-Asp453 | HEK293 | P15423.1 | 19 |
| HCoV-NL63 | S1 domain | Sino Biological | 40600-V08H | Cys19-Val717 | HEK293 | APF29071.1 | 78.8 |
| HCoV-NL63 | RBD domain | Recombinant expression in-house | – | 481-616 | HEK293 | QE59359.1 | 21.1 |
| – | Human serum albumin | Merck Sigma-Aldrich® (Purified) | A9511-500mg | – | – | – | 66 |
| – | Goat Anti-human IgG (Fc) | Dianova (Purified, MinX) | 109-005-008 | – | – | – | 150 |
| – | Pertussis toxin | List Labs (Purified, Salt-free) | #181 | – | – | – | 117 |

**Table S2.** Cut-off values (with 95% confidence interval) for SARS-CoV-2-specific antigens in the bead-based multiplex assay developed in this work when determined by a population-based cut-off method (first line) or by method comparison with different assays used as reference assays (lines 2-8).

|  | TriS | lower | upper | S1 | lower | upper | RBD | lower | upper | N | lower | upper |
| --- | --- | --- | --- | --- | --- | --- | --- | --- | --- | --- | --- | --- |
| Population-based | **2.72** | **2.70** | **2.74** | **2.22** | **2.21** | **2.23** | **2.39** | **2.37** | **2.41** | **2.70** | **2.69** | **2.72** |
| VNT | 3.04 | 3.00 | 3.13 | 2.90 | 2.86 | 2.94 | 2.85 | 2.82 | 2.87 | 2.90 | 2.71 | 2.96 |
| sVNT | 3.13 | 3.03 | 3.15 | 2.96 | 2.87 | 3.02 | 2.92 | 2.85 | 2.97 | 2.93 | 2.87 | 2.99 |
| EUROIMMUN-ELISA S | **2.80** | **2.69** | **2.96** | **2.70** | **2.63** | **2.75** | **2.64** | **2.55** | **2.70** | 2.47 | 2.31 | 2.75 |
| DiaSorin-CLIACLIA S | **3.00** | **2.87** | **3.04** | **2.78** | **2.75** | **2.86** | **2.70** | **2.68** | **2.85** | 2.75 | 2.59 | 2.87 |
| Roche-ECLIA_S | **2.68** | **2.50** | **2.84** | **2.26** | **2.15** | **2.30** | **2.36** | **2.17** | **2.43** | 2.39 | 2.28 | 2.73 |
| Roche-ECLIA_N | 2.79 | 2.53 | 2.86 | 2.26 | 2.18 | 2.77 | 2.36 | 2.17 | 2.43 | **2.49** | **2.34** | **2.73** |
| Abbott-CMIA N | 3.10 | 3.04 | 3.15 | 2.99 | 2.87 | 3.02 | 2.91 | 2.84 | 2.98 | 2.93 | 2.90 | 2.96 |
| Median^1^ | 2.76 | 2.70 | 2.90 | 2.48 | 2.42 | 2.53 | 2.51 | 2.46 | 2.57 | 2.59 | 2.52 | 2.72 |

^1^ Median values as calculated from bold values above (Population-based, EUROIMMUN-ELISA S, DiaSorin-CLIA S, Roche-ECLIA S für S-specific antigens; Population-based and Roche-ECLIA N for N-specific antigen.).

**Table S3.** Results for method comparison between all antigens implemented in the 17-plex suspension assay established in this work and the combined SARS-CoV-2-specific antigens (TriS, S1, RBD, and N) and the tested reference assays: VNT, Abbott-CMIA N, DiaSorin-CLIA S, EUROIMMUN-ELISA S, sVNT, Roche-ECLIA S, and Roche-ECLIA N.

| Reference assay | Test assay/antigen | Number of sera | Cutoff | Accuracy (%) | Sensitivity (%) | Specificity (%) | Positive predictive value (%) | Negative predictive value (%) |
| --- | --- | --- | --- | --- | --- | --- | --- | --- |
| VNT | Abbott-CMIA N | 464 | 1.235 | 89.7 | 90.3 | 88.3 | 93.8 | 82 |
| VNT | ahIgG | 495 | 14334.5 | 55.8 | 55.2 | 56.2 | 70.9 | 39.6 |
| VNT | Combined | 495 | 0.794 | 95.2 | 95.4 | 94.7 | 97.2 | 91.5 |
| VNT | DiaSorin-CLIA S | 464 | 20.65 | 93.1 | 93.2 | 93.5 | 96.7 | 87 |
| VNT | EUROIMMUN-ELISA S | 495 | 1.663 | 91.7 | 92 | 91.1 | 95.3 | 85.4 |
| VNT | sVNT | 483 | 29.174 | 94.6 | 93.8 | 96.2 | 98.1 | 88.6 |
| VNT | HCoV-229E RBD | 495 | 680.5 | 54.1 | 56.4 | 49.1 | 68.1 | 37 |
| VNT | HCoV-229E S1 | 495 | 5433.5 | 55.4 | 55.2 | 55 | 70.2 | 38.9 |
| VNT | HCoV-HKU1 RBD | 495 | 3276 | 52.5 | 51.5 | 55 | 68.6 | 36.9 |
| VNT | HCoV-HKU1 S1 | 494 | 2708.25 | 52.9 | 51.2 | 55.6 | 69.1 | 37.3 |
| VNT | HCoV-NL63 RBD | 495 | 899 | 53.3 | 54.3 | 49.7 | 67.8 | 36.4 |
| VNT | HCoV-NL63 S1 | 494 | 1795.5 | 54.5 | 55.8 | 53.3 | 69.5 | 38 |
| VNT | HCoV-OC43 RBD | 494 | 6854.5 | 52.7 | 52.6 | 52.7 | 68.8 | 37 |
| VNT | HCoV-OC43 S1 | 495 | 4053 | 54.5 | 52.5 | 59.2 | 71.3 | 39.1 |
| VNT | HSA | 495 | 106.5 | 57 | 63.2 | 45.6 | 68.9 | 38.7 |
| VNT | MERS-CoV S1 | 495 | 69.5 | 53.5 | 55.2 | 49.7 | 68 | 36.6 |
| VNT | Pertussistoxin | 495 | 2889 | 53.9 | 52.1 | 56.8 | 70 | 38.2 |
| VNT | Roche-ECLIA N | 127 | 14.21 | 88.2 | 89.6 | 86.7 | 88.1 | 88.1 |
| VNT | Roche-ECLIA S | 127 | 24.285 | 91.3 | 91 | 91.7 | 92.4 | 90.3 |
| VNT | SARS-CoV-2 N | 495 | 2.896 | 88.9 | 90.2 | 86.4 | 92.7 | 81.9 |
| VNT | SARS-CoV-2 RBD | 495 | 2.845 | 94.7 | 95.4 | 93.5 | 96.6 | 91.5 |
| VNT | SARS-CoV-2 S1 | 495 | 2.902 | 93.7 | 94.5 | 92.3 | 95.9 | 89.5 |
| VNT | SARS-CoV-2 TriS | 495 | 3.044 | 91.7 | 92.6 | 90.5 | 94.8 | 86.3 |
| VNT | SARS-CoV S1 | 495 | 216 | 86.1 | 85 | 87.6 | 93.1 | 75.1 |
| Abbott-CMIA N | Abbott-CMIA N | 486 | 1.37 | 100 | 100 | 100 | 100 | 100 |
| Abbott-CMIA N | ahIgG | 485 | 14328.5 | 53.1 | 54 | 51.8 | 62.5 | 43 |
| Abbott-CMIA N | Combined | 486 | 0.691 | 96.3 | 96.9 | 95.9 | 97.2 | 95.3 |
| Abbott-CMIA N | DiaSorin-CLIA S | 486 | 23.8 | 89 | 89.7 | 88.2 | 91.8 | 85.1 |
| Abbott-CMIA N | EUROIMMUN-ELISA S | 486 | 2.141 | 88.5 | 88.3 | 88.7 | 92.3 | 83.6 |
| Abbott-CMIA N | sVNT | 477 | 39.289 | 86.2 | 85.9 | 86.6 | 90.8 | 79.8 |
| Abbott-CMIA N | HCoV-229E RBD | 485 | 692 | 52.1 | 51.5 | 53.3 | 62 | 42.3 |
| Abbott-CMIA N | HCoV-229E S1 | 485 | 5217.5 | 55.6 | 53.3 | 57.9 | 65.6 | 45.7 |
| Abbott-CMIA N | HCoV-HKU1 RBD | 486 | 3246.5 | 53.7 | 53.6 | 53.8 | 63.7 | 43.9 |
| Abbott-CMIA N | HCoV-HKU1 S1 | 486 | 3074.25 | 53.7 | 50.9 | 55.4 | 63.4 | 43.8 |
| Abbott-CMIA N | HCoV-NL63 RBD | 485 | 861.75 | 52.3 | 51.5 | 52.3 | 62 | 42.3 |
| Abbott-CMIA N | HCoV-NL63 S1 | 485 | 1891.5 | 52.1 | 50.5 | 54.4 | 62.2 | 42.4 |
| Abbott-CMIA N | HCoV-OC43 RBD | 486 | 7188.5 | 51.6 | 53.6 | 49.2 | 61.2 | 41.5 |
| Abbott-CMIA N | HCoV-OC43 S1 | 485 | 3740.75 | 54.7 | 50.9 | 60.5 | 65.8 | 45.3 |
| Abbott-CMIA N | HSA | 485 | 98.5 | 53.7 | 56.5 | 49.7 | 62.6 | 43.3 |
| Abbott-CMIA N | MERS-CoV S1 | 486 | 69.75 | 53.5 | 56.4 | 49.2 | 62.4 | 43 |
| Abbott-CMIA N | Pertussistoxin | 485 | 2883.25 | 54.5 | 54 | 54.4 | 64 | 44.4 |
| Abbott-CMIA N | Roche-ECLIA N | 127 | 15.215 | 97.6 | 97.1 | 98.3 | 98.5 | 96.6 |
| Abbott-CMIA N | Roche-ECLIA S | 127 | 20.7 | 87.4 | 89.9 | 84.5 | 87.7 | 87.3 |
| Abbott-CMIA N | SARS-CoV-2 N | 486 | 2.933 | 96.5 | 96.9 | 95.9 | 97.2 | 95.4 |
| Abbott-CMIA N | SARS-CoV-2 RBD | 486 | 2.921 | 89.3 | 91.1 | 86.7 | 91.1 | 86.5 |
| Abbott-CMIA N | SARS-CoV-2 S1 | 486 | 2.987 | 89.1 | 90 | 87.7 | 91.7 | 85.5 |
| Abbott-CMIA N | SARS-CoV-2 TriS | 486 | 3.103 | 88.1 | 90.4 | 84.6 | 89.8 | 85.6 |
| Abbott-CMIA N | SARS-CoV S1 | 486 | 244.125 | 83.1 | 82.1 | 84.1 | 88.5 | 76 |
| DiaSorin-CLIA S | Abbott-CMIA N | 486 | 1,075 | 92 | 91.7 | 92.5 | 96.2 | 84.7 |
| DiaSorin-CLIA S | ahIgG | 486 | 14346.5 | 54.3 | 54.6 | 53.1 | 70.4 | 36.6 |
| DiaSorin-CLIA S | Combined | 486 | 0.692 | 96.7 | 96.9 | 96.2 | 98.1 | 93.9 |
| DiaSorin-CLIA S | DiaSorin-CLIA S | 486 | 15 | 100 | 100 | 100 | 100 | 100 |
| DiaSorin-CLIA S | EUROIMMUN-ELISA S | 486 | 1.349 | 95.7 | 96.6 | 94.4 | 97.1 | 93 |
| DiaSorin-CLIA S | sVNT | 478 | 18.835 | 94.1 | 94.2 | 94.1 | 97.2 | 88.3 |
| DiaSorin-CLIA S | HCoV-229E RBD | 486 | 645.25 | 54.3 | 57.1 | 48.8 | 69.3 | 35.7 |
| DiaSorin-CLIA S | HCoV-229E S1 | 485 | 5339.25 | 55.1 | 53.1 | 58.8 | 72.4 | 38.2 |
| DiaSorin-CLIA S | HCoV-HKU1 RBD | 486 | 3249.5 | 51.9 | 50.3 | 55.6 | 69.7 | 35.3 |
| DiaSorin-CLIA S | HCoV-HKU1 S1 | 486 | 2732.75 | 52.9 | 51.5 | 56.2 | 70.4 | 36.1 |
| DiaSorin-CLIA S | HCoV-NL63 RBD | 486 | 861.75 | 52.7 | 51.8 | 52.5 | 69.3 | 35.3 |
| DiaSorin-CLIA S | HCoV-NL63 S1 | 486 | 2026 | 51.9 | 50 | 54.4 | 69.1 | 34.9 |
| DiaSorin-CLIA S | HCoV-OC43 RBD | 486 | 7365.75 | 53.3 | 54.6 | 51.2 | 69.3 | 35.4 |
| DiaSorin-CLIA S | HCoV-OC43 S1 | 486 | 4050 | 57 | 55.8 | 59.4 | 73.6 | 39.7 |
| DiaSorin-CLIA S | HSA | 486 | 96.5 | 56.2 | 61 | 46.2 | 69.9 | 36.9 |
| DiaSorin-CLIA S | MERS-CoV S1 | 486 | 69.5 | 54.1 | 55.8 | 50 | 69.6 | 35.9 |
| DiaSorin-CLIA S | Pertussistoxin | 485 | 3368.75 | 54.1 | 54.9 | 52.5 | 70.3 | 36.5 |
| DiaSorin-CLIA S | Roche-ECLIA N | 127 | 10.835 | 93.7 | 94.6 | 92.5 | 94.6 | 92.5 |
| DiaSorin-CLIA S | Roche-ECLIA S | 127 | 19.765 | 94.5 | 93.2 | 96.2 | 97.2 | 91.1 |
| DiaSorin-CLIA S | SARS-CoV-2 N | 486 | 2.746 | 92.8 | 92.9 | 92.5 | 96.2 | 86.6 |
| DiaSorin-CLIA S | SARS-CoV-2 RBD | 486 | 2.703 | 96.5 | 97.9 | 94.4 | 97.2 | 95.5 |
| DiaSorin-CLIA S | SARS-CoV-2 S1 | 486 | 2.784 | 95.7 | 96.6 | 93.8 | 96.9 | 93.2 |
| DiaSorin-CLIA S | SARS-CoV-2 TriS | 486 | 3,002 | 95.9 | 96.3 | 95.6 | 97.8 | 92.6 |
| DiaSorin-CLIA S | SARS-CoV S1 | 486 | 159.5 | 90.3 | 90.8 | 89.4 | 94.5 | 82.6 |
| EUROIMMUN-ELISA S | Abbott-CMIA N | 467 | 0.405 | 95.7 | 96.4 | 94.6 | 97.8 | 91 |
| EUROIMMUN-ELISA S | ahIgG | 502 | 14343.25 | 55.3 | 54.9 | 55.4 | 74.8 | 34 |
| EUROIMMUN-ELISA S | Combined | 503 | 1.869 | 97.2 | 96.9 | 98 | 99.1 | 92.9 |
| EUROIMMUN-ELISA S | DiaSorin-CLIA S | 467 | 11.9 | 97.4 | 96.7 | 99.2 | 99.7 | 92.1 |
| EUROIMMUN-ELISA S | EUROIMMUN-ELISA S | 503 | 0.955 | 100 | 100 | 100 | 100 | 100 |
| EUROIMMUN-ELISA S | sVNT | 491 | 10.967 | 94.7 | 94.1 | 97.1 | 98.8 | 86.3 |
| EUROIMMUN-ELISA S | HCoV-229E RBD | 502 | 677.75 | 54.1 | 55.2 | 51.4 | 73 | 32.2 |
| EUROIMMUN-ELISA S | HCoV-229E S1 | 503 | 5252.75 | 53.9 | 51 | 59.5 | 75.3 | 33.9 |
| EUROIMMUN-ELISA S | HCoV-HKU1 RBD | 503 | 3917.75 | 50.9 | 45.9 | 60.8 | 73.8 | 32.2 |
| EUROIMMUN-ELISA S | HCoV-HKU1 S1 | 503 | 2761 | 51.1 | 50.4 | 53.4 | 72.1 | 30.9 |
| EUROIMMUN-ELISA S | HCoV-NL63 RBD | 503 | 861.75 | 52.1 | 51 | 54.1 | 72.9 | 31.7 |
| EUROIMMUN-ELISA S | HCoV-NL63 S1 | 503 | 1815.5 | 53.9 | 55.8 | 49.3 | 72.6 | 31.7 |
| EUROIMMUN-ELISA S | HCoV-OC43 RBD | 503 | 6250.25 | 54.9 | 58.3 | 47.3 | 72.5 | 32 |
| EUROIMMUN-ELISA S | HCoV-OC43 S1 | 502 | 4053 | 55.2 | 54.6 | 56.8 | 75 | 34.1 |
| EUROIMMUN-ELISA S | HSA | 503 | 98.25 | 55.7 | 58.6 | 48.6 | 73.1 | 32.7 |
| EUROIMMUN-ELISA S | MERS-CoV S1 | 503 | 71.25 | 54.7 | 56.9 | 47.3 | 72.7 | 32.2 |
| EUROIMMUN-ELISA S | Pertussistoxin | 502 | 2883.25 | 53.7 | 51.5 | 58.4 | 74.8 | 33.6 |
| EUROIMMUN-ELISA S | Roche-ECLIA N | 121 | 3.41 | 95 | 94.9 | 95.2 | 97.3 | 91.1 |
| EUROIMMUN-ELISA S | Roche-ECLIA S | 121 | 5.66 | 95.9 | 96.2 | 95.2 | 97.5 | 93.2 |
| EUROIMMUN-ELISA S | SARS-CoV-2 N | 503 | 2.493 | 94.6 | 95.5 | 93.2 | 97 | 89.4 |
| EUROIMMUN-ELISA S | SARS-CoV-2 RBD | 503 | 2.641 | 96.8 | 96.3 | 98 | 99.1 | 91.8 |
| EUROIMMUN-ELISA S | SARS-CoV-2 S1 | 503 | 2.698 | 97.8 | 97.5 | 98 | 99.1 | 94.3 |
| EUROIMMUN-ELISA S | SARS-CoV-2 TriS | 503 | 2.804 | 96 | 96.3 | 95.3 | 98 | 91.7 |
| EUROIMMUN-ELISA S | SARS-CoV S1 | 503 | 132 | 90.7 | 90.7 | 90.5 | 95.9 | 80.1 |
| sVNT | Abbott-CMIA N | 478 | 1.29 | 87 | 89.2 | 84.1 | 90 | 82.6 |
| sVNT | ahIgG | 511 | 14317 | 54.5 | 53.1 | 56.6 | 64.7 | 44.7 |
| sVNT | Combined | 512 | 0.647 | 93.4 | 93.5 | 93.2 | 95.5 | 90.4 |
| sVNT | DiaSorin-CLIA S | 478 | 25.45 | 92.7 | 91.9 | 94 | 96.1 | 87.8 |
| sVNT | EUROIMMUN-ELISA S | 512 | 2,035 | 91.6 | 91.5 | 91.7 | 94.3 | 88.1 |
| sVNT | sVNT | 512 | 30.2 | 100 | 100 | 100 | 100 | 100 |
| sVNT | HCoV-229E RBD | 511 | 831.5 | 55.1 | 61.2 | 47.3 | 63.3 | 44.4 |
| sVNT | HCoV-229E S1 | 511 | 5316.25 | 56.6 | 55 | 58.5 | 66.5 | 46.6 |
| sVNT | HCoV-HKU1 RBD | 511 | 3249.5 | 53.3 | 52.1 | 55.1 | 63.5 | 43.5 |
| sVNT | HCoV-HKU1 S1 | 511 | 2708.25 | 53.1 | 51.1 | 56.1 | 63.5 | 43.4 |
| sVNT | HCoV-NL63 RBD | 512 | 895.5 | 55.1 | 55.7 | 52.7 | 64.1 | 44.8 |
| sVNT | HCoV-NL63 S1 | 512 | 1799 | 53.7 | 56.7 | 49.8 | 62.8 | 43.3 |
| sVNT | HCoV-OC43 RBD | 511 | 6894 | 51.8 | 50.5 | 54.1 | 62.1 | 42 |
| sVNT | HCoV-OC43 S1 | 512 | 3610 | 54.5 | 50.2 | 61 | 65.7 | 45 |
| sVNT | HSA | 512 | 98.25 | 54.3 | 58.6 | 47.3 | 62.5 | 43.4 |
| sVNT | MERS-CoV S1 | 512 | 69.75 | 52.7 | 55.7 | 48.3 | 61.8 | 42.2 |
| sVNT | Pertussistoxin | 511 | 2883.5 | 55.3 | 53.4 | 57.1 | 65.2 | 45.3 |
| sVNT | Roche-ECLIA N | 127 | 16.44 | 85.8 | 87.9 | 83.6 | 85.1 | 86.7 |
| sVNT | Roche-ECLIA S | 127 | 24.285 | 90.6 | 89.4 | 90.2 | 91.2 | 89.2 |
| sVNT | SARS-CoV-2 N | 512 | 2.931 | 87.7 | 89.6 | 84.9 | 89.9 | 84.4 |
| sVNT | SARS-CoV-2 RBD | 512 | 2.92 | 92.4 | 92.5 | 92.2 | 94.7 | 89.2 |
| sVNT | SARS-CoV-2 S1 | 512 | 2.959 | 92.4 | 93.2 | 91.2 | 94.2 | 90 |
| sVNT | SARS-CoV-2 TriS | 512 | 3.133 | 91 | 90.9 | 90.7 | 93.5 | 86.9 |
| sVNT | SARS-CoV S1 | 512 | 217.75 | 87.3 | 87 | 87.8 | 91.5 | 81.7 |
| Roche-ECLIA N | Abbott-CMIA N | 127 | 0.615 | 99.2 | 100 | 97.6 | 98.9 | 100 |
| Roche-ECLIA N | ahIgG | 126 | 14290 | 60.6 | 64 | 56.1 | 75 | 41.6 |
| Roche-ECLIA N | Combined | 127 | -0.331 | 100 | 100 | 100 | 100 | 100 |
| Roche-ECLIA N | DiaSorin-CLIA S | 127 | 8.34 | 98.4 | 98.8 | 97.6 | 98.9 | 97.6 |
| Roche-ECLIA N | EUROIMMUN-ELISA S | 127 | 0.892 | 96.1 | 96.5 | 95.1 | 97.6 | 92.7 |
| Roche-ECLIA N | sVNT | 127 | 8.133 | 97.6 | 97.7 | 100 | 100 | 95.2 |
| Roche-ECLIA N | HCoV-229E RBD | 127 | 716.5 | 57.5 | 60.5 | 51.2 | 72.3 | 38.2 |
| Roche-ECLIA N | HCoV-229E S1 | 125 | 4928 | 57.5 | 59.3 | 56.1 | 73.6 | 39.1 |
| Roche-ECLIA N | HCoV-HKU1 RBD | 127 | 2444 | 60.6 | 64 | 53.7 | 74.3 | 41.5 |
| Roche-ECLIA N | HCoV-HKU1 S1 | 127 | 2000.75 | 60.6 | 64 | 53.7 | 74.3 | 41.9 |
| Roche-ECLIA N | HCoV-NL63 RBD | 126 | 906 | 56.7 | 59.3 | 51.2 | 71.9 | 37.7 |
| Roche-ECLIA N | HCoV-NL63 S1 | 127 | 1546 | 61.4 | 64 | 58.5 | 76.4 | 43.3 |
| Roche-ECLIA N | HCoV-OC43 RBD | 126 | 6250.25 | 55.9 | 57 | 56.1 | 72.6 | 37.8 |
| Roche-ECLIA N | HCoV-OC43 S1 | 126 | 4003.5 | 56.7 | 58.1 | 56.1 | 72.7 | 38.1 |
| Roche-ECLIA N | HSA | 127 | 96.5 | 60.6 | 64 | 51.2 | 73.5 | 41 |
| Roche-ECLIA N | MERS-CoV S1 | 127 | 84.25 | 62.2 | 69.8 | 46.3 | 73.3 | 42.1 |
| Roche-ECLIA N | Pertussistoxin | 127 | 3020 | 56.7 | 54.7 | 58.5 | 74 | 38.8 |
| Roche-ECLIA N | Roche-ECLIA N | 127 | 1.236 | 100 | 100 | 100 | 100 | 100 |
| Roche-ECLIA N | Roche-ECLIA S | 127 | 2.975 | 99.2 | 100 | 97.6 | 98.9 | 100 |
| Roche-ECLIA N | SARS-CoV-2 N | 127 | 2.487 | 98.4 | 100 | 97.6 | 98.9 | 100 |
| Roche-ECLIA N | SARS-CoV-2 RBD | 127 | 2.359 | 99.2 | 100 | 97.6 | 98.9 | 100 |
| Roche-ECLIA N | SARS-CoV-2 S1 | 127 | 2.262 | 98.4 | 100 | 97.6 | 98.8 | 100 |
| Roche-ECLIA N | SARS-CoV-2 TriS | 127 | 2.792 | 98.4 | 98.8 | 97.6 | 98.8 | 97.6 |
| Roche-ECLIA N | SARS-CoV S1 | 127 | 89.5 | 85 | 87.2 | 80.5 | 90.5 | 75.6 |
| Roche-ECLIA S | Abbott-CMIA N | 127 | 0.615 | 98.4 | 100 | 97.5 | 98.9 | 100 |
| Roche-ECLIA S | ahIgG | 127 | 14290 | 61.4 | 64.4 | 57.5 | 76.4 | 41.5 |
| Roche-ECLIA S | Combined | 127 | -0.331 | 100 | 100 | 100 | 100 | 100 |
| Roche-ECLIA S | DiaSorin-CLIA S | 127 | 8.34 | 99.2 | 98.9 | 100 | 100 | 97.6 |
| Roche-ECLIA S | EUROIMMUN-ELISA S | 127 | 0.818 | 96.9 | 97.7 | 95 | 97.7 | 94.9 |
| Roche-ECLIA S | sVNT | 127 | -0.224 | 98.4 | 100 | 97.5 | 98.9 | 100 |
| Roche-ECLIA S | HCoV-229E RBD | 126 | 716.5 | 56.7 | 59.8 | 52.5 | 72.4 | 36.2 |
| Roche-ECLIA S | HCoV-229E S1 | 127 | 4928 | 56.7 | 57.5 | 55 | 73.8 | 37.5 |
| Roche-ECLIA S | HCoV-HKU1 RBD | 127 | 2076.5 | 62.2 | 66.7 | 52.5 | 75 | 41.7 |
| Roche-ECLIA S | HCoV-HKU1 S1 | 126 | 1775 | 62.2 | 67.8 | 50 | 75 | 42.3 |
| Roche-ECLIA S | HCoV-NL63 RBD | 126 | 906 | 57.5 | 59.8 | 52.5 | 73.4 | 37.7 |
| Roche-ECLIA S | HCoV-NL63 S1 | 127 | 1546 | 60.6 | 62.1 | 60 | 76.5 | 41.4 |
| Roche-ECLIA S | HCoV-OC43 RBD | 127 | 6250.25 | 55.1 | 55.2 | 55 | 72.9 | 36.1 |
| Roche-ECLIA S | HCoV-OC43 S1 | 126 | 4003.5 | 57.5 | 58.6 | 57.5 | 74.3 | 38.2 |
| Roche-ECLIA S | HSA | 127 | 96.5 | 60.6 | 63.2 | 52.5 | 75 | 40.7 |
| Roche-ECLIA S | MERS-CoV S1 | 127 | 84.25 | 62.2 | 70.1 | 45 | 73.7 | 40.9 |
| Roche-ECLIA S | Pertussistoxin | 127 | 3020 | 57.5 | 55.2 | 62.5 | 75.7 | 38.8 |
| Roche-ECLIA S | Roche-ECLIA N | 127 | 0.493 | 100 | 100 | 100 | 100 | 100 |
| Roche-ECLIA S | Roche-ECLIA S | 127 | 2.975 | 100 | 100 | 100 | 100 | 100 |
| Roche-ECLIA S | SARS-CoV-2 N | 127 | 2.391 | 98.4 | 100 | 97.5 | 98.9 | 100 |
| Roche-ECLIA S | SARS-CoV-2 RBD | 127 | 2.359 | 100 | 100 | 100 | 100 | 100 |
| Roche-ECLIA S | SARS-CoV-2 S1 | 127 | 2.262 | 99.2 | 100 | 97.5 | 98.9 | 100 |
| Roche-ECLIA S | SARS-CoV-2 TriS | 127 | 2.675 | 98.4 | 100 | 97.5 | 98.9 | 100 |
| Roche-ECLIA S | SARS-CoV S1 | 127 | 89.5 | 86.6 | 88.5 | 82.5 | 91.4 | 76.7 |

**Table S4.** Seroconversion (%) in patients (n) after immunisation with the Astra Zeneca or BioNTech vaccine or infection with the Alpha (B.1.1.7) or Beta (B.1.351) variant as determined by the SARS-CoV-2-specific antigens implemented in the bead-based multiplex assay.

| Group | Time point/Variant | Patients (n) | SARS-CoV-2 S1 | SARS-CoV-2 TriS | SARS-CoV-2 RBD | SARS-CoV-2 N |
| --- | --- | --- | --- | --- | --- | --- |
| Astra Zeneca | pre | 19 | 0 | 0 | 0 | 0 |
|  | pre (infected) | 1 | 100 | 100 | 100 | 100 |
|  | 1st | 20 | 90 | 80 | 85 | 10 |
| BioNTech | pre | 18 | 0 | 0 | 0 | 0 |
|  | pre (infected) | 2 | 100 | 100 | 100 | 50 |
|  | 1st | 20 | 100 | 95 | 95 | 0 |
|  | 2nd | 20 | 100 | 100 | 100 | 0 |
| Infected | Alpha (B.1.1.7) | 20 | 90 | 90 | 85 | 90 |
|  | Beta (B.1.351) | 16 | 75 | 87.5 | 62.5 | 100 |

# Supporting references

1 von Berg, L. *et al.* Functional detection of botulinum neurotoxin serotypes A to F by monoclonal neoepitope-specific antibodies and suspension array technology. *Sci Rep* **9**, 5531 (2019).

2 Laemmli, U. K. Cleavage of structural proteins during the assembly of the head of bacteriophage T4. *Nature* **227**, 680-685 (1970).

3 Neuhoff, V., Arold, N., Taube, D. & Ehrhardt, W. Improved staining of proteins in polyacrylamide gels including isoelectric focusing gels with clear background at nanogram sensitivity using Coomassie Brilliant Blue G-250 and R-250. *Electrophoresis* **9**, 255-262 (1988).
